# Cytoplasmic and mitochondrial aminoacyl-tRNA synthetases differentially regulate lifespan in *Caenorhabditis elegans*

**DOI:** 10.1016/j.isci.2022.105266

**Published:** 2022-10-03

**Authors:** Tianlin Zheng, Qiang Luo, Chengxuan Han, Jiejun Zhou, Jianke Gong, Lei Chun, X.Z. Shawn Xu, Jianfeng Liu

**Affiliations:** 1 College of Life Science and Technology, Key Laboratory of Molecular Biophysics of MOE, Huazhong University of Science and Technology, Wuhan, Hubei 430074, China; 2 Life Sciences Institute and Department of Molecular and Integrative Physiology, University of Michigan, Ann Arbor, MI 48109, USA

**Keywords:** Molecular biology, Cell biology

## Abstract

Reducing the rate of translation promotes longevity in multiple organisms, representing a conserved mechanism for lifespan extension. Aminoacyl-tRNA synthetases (ARSs) catalyze the loading of amino acids to their cognate tRNAs, thereby playing an essential role in translation. Mutations in ARS genes are associated with various human diseases. However, little is known about the role of ARSs in aging, particularly whether and how these genes regulate lifespan. Here, using *Caenorhabditis elegans* as a model, we systematically characterized the role of all three types of ARS genes in lifespan regulation, including mitochondrial, cytoplasmic, and cyto-mito bifunctional ARS genes. We found that, as expected, RNAi knockdown of mitochondrial ARS genes extended lifespan. Surprisingly, knocking down cytoplasmic or cyto-mito bifunctional ARS genes shortened lifespan, though such treatment reduced the rate of translation. These results reveal opposing roles of mitochondrial and cytoplasmic ARSs in lifespan regulation, demonstrating that inhibiting translation may not always extend lifespan.

## Highlights

• RNAi knockdown of mitochondrial ARS genes extends lifespan via UPR^mt^
• RNAi knockdown of cytoplasmic or cyto-mito bifunctional ARS genes shortens lifespan
• Inhibiting translation may not always extend lifespan

## Graphical abstract

**Figure:**
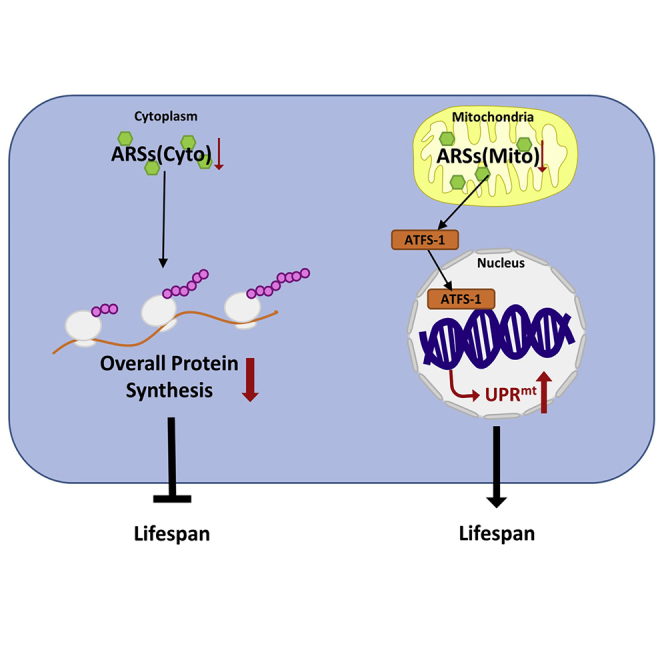

## Introduction

mRNA translation is a highly regulated multi-step process (Sonenberg and Hinnebusch, 2007). Inhibiting translation slows down growth and development, yet paradoxically extends lifespan in multiple organisms, including yeast, worms and flies (Chen et al., 2007; Ching et al., 2010; Chiocchetti et al., 2007; Curran and Ruvkun, 2007; Demontis and Perrimon, 2010; Hansen et al., 2007; Heissenberger et al., 2020; Hu et al., 2018; Pan et al., 2007; Rogers et al., 2011; Schosserer et al., 2015; Steffen et al., 2008; Syntichaki et al., 2007; Tiku et al., 2017; Xie et al., 2019). For example, reducing the expression or inhibiting the function of various components in the translation machinery, such as translation initiation factors (Chen et al., 2007; Ching et al., 2010; Curran and Ruvkun, 2007; Hansen et al., 2007; Pan et al., 2007; Rogers et al., 2011; Syntichaki et al., 2007), elongation factors (Xie et al., 2019), ribosomal RNAs (Essers et al., 2015; Heissenberger et al., 2020; Schosserer et al., 2015; Tiku et al., 2017), and ribosomal proteins (Chen et al., 2007; Chiocchetti et al., 2007; Curran and Ruvkun, 2007; Hansen et al., 2007; Houtkooper et al., 2013; Molenaars et al., 2020), all promote longevity. Although the underlying mechanisms remain unclear, it has been suggested that inhibiting translation may selectively increase the relative expression levels of stress response genes that promote somatic maintenance, thereby extending lifespan (Rogers et al., 2011).

tRNA charging is a crucial step in translation, which is catalyzed by aminoacyl-tRNA synthetases (ARSs) (Antonellis and Green, 2008). These enzymes are ubiquitously expressed and are responsible for loading amino acids to their cognate tRNAs in the cytoplasm and mitochondria (Antonellis and Green, 2008; Kuo and Antonellis, 2020). Each amino acid has its specific ARS, with the exception of glutamine in mitochondrial proteins. In human mitochondria, the glutamyl-tRNA synthetase charges tRNA^Gln^ as well as tRNA^Glu^ with glutamate. A second enzyme, GatCAB aminoacyl-tRNA amidotransferase, then converts Glu-tRNA^Gln^ to Gln-tRNA^Gln^ (Friederich et al., 2018; Nagao et al., 2009). Most ARSs have evolved into separate genes which encode cytoplasm-specific or mitochondria-specific ARSs, producing charged tRNAs for mRNA translation in the cytoplasm and mitochondria, respectively. Notably, a few ARS genes are bifunctional (Antonellis and Green, 2008), encoding both cytoplasmic and mitochondrial isoforms via alternative translation initiation (Chihara et al., 2007) or alternative splicing (Tolkunova et al., 2000). Cytoplasmic ARSs are responsible for the synthesis of the vast majority of proteins in the cell, whereas mitochondrial ARSs merely mediate the synthesis of 12 or 13 proteins that participate in oxidative phosphorylation (Mishra and Chan, 2014). Dysfunction of ARSs suppresses cytoplasmic and/or mitochondrial protein synthesis, and mutations in several ARS loci lead to a variety of human diseases, including peripheral neuropathies (Antonellis et al., 2003; Jordanova et al., 2006; Latour et al., 2010; Tsai et al., 2017; Vester et al., 2013), sensorineural hearing loss (Santos-Cortez et al., 2013; Zhou et al., 2017), infantile liver diseases (Casey et al., 2012, 2015; Peroutka et al., 2019), and brittle hair and nails (Kuo et al., 2019; Theil et al., 2019). Nevertheless, despite their essential role in translation, little is known about the role of ARSs in aging.

In this study, we systematically examined the role of ARS genes in lifespan regulation in *Caenorhabditis elegans*, a genetic model organism widely used for aging research (Kenyon, 2010). We first characterized a mutant allele of *ears-2* gene that encodes the mitochondrial glutamyl-tRNA synthetase, and found that *ears-2* mutant worms or worms with *ears-2* knocked down by RNAi are long-lived. This long-lived phenotype is mediated by UPR^mt^. Much to our surprise, knockdown of *ears-1* gene, which encodes the cytoplasmic homolog of *ears-2*, substantially shortened lifespan, though the rate of translation is reduced in these worms. We then characterized all the remaining ARS genes in the *C*. *elegans* genome, and found that RNAi of mitochondria-specific ARS genes all extended lifespan, whereas RNAi of cytoplasm-specific or cyto-mito bifunctional ARS genes all shortened lifespan. These results identify opposing roles of mitochondrial and cytoplasmic ARSs in lifespan regulation, revealing the rather complex nature of translation in aging. We suggest that global suppression of cytoplasmic protein synthesis is detrimental to animals, leading to shortened lifespan. Thus, reducing the rate of translation may not always promote longevity.

## Results

### *ears-2* mutant worms are long-lived

**Figure 1:**
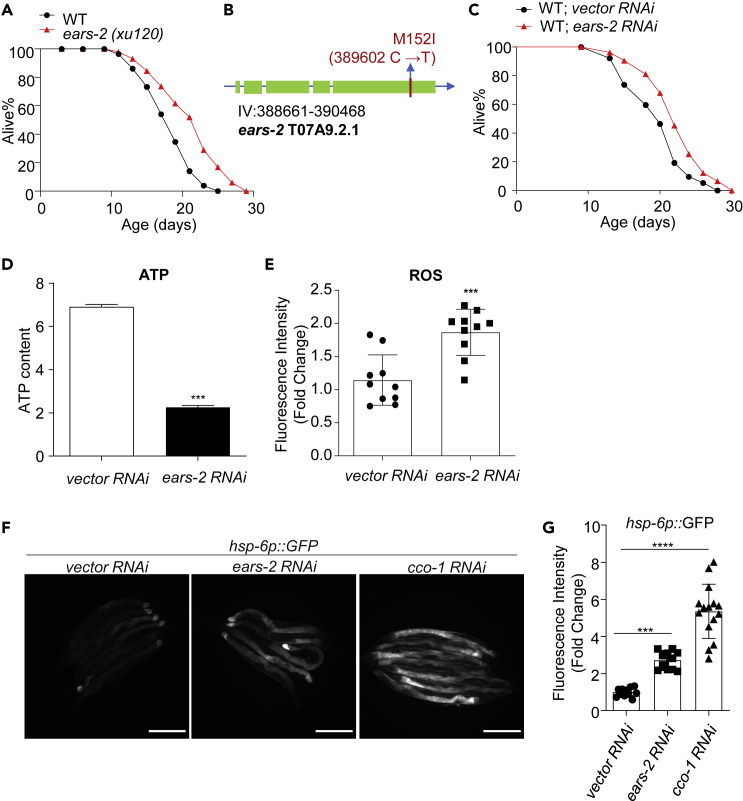
Inactivation of *ears-2* extends lifespan and alters mitochondrial functions (A) *ears-2(xu120)* mutant worms are long-lived. (B) Genetic mapping of the *ears-2(xu120)* mutant allele. (C) *ears-2* RNAi extends lifespan. All lifespan assays were performed at 20°C and were repeated at least twice. See Table S1 for lifespan statistics. Logrank (Kaplan-Meier) was used to calculate p values. (D) *ears-2* RNAi decreases the ATP level. The same number (∼120) of day1 adult worms treated by feeding RNAi were used for ATP level test. n = 3. ∗∗∗p< 0.001 (t test). (E) *ears-2* RNAi enhances the ROS level. 10 μMol/L DCFH-DA was used for ROS measurement. n = 10. ∗∗∗p< 0.001 (t test). (F–G) *ears-2* RNAi induces UPR^mt^, similar to the effect of *cco-1* RNAi. Representative images (F) and quantification graph (G) are shown. Scale bars, 300 μm. n = 11–15. Error bars represent SE of mean. ∗∗∗p< 0.001, ∗∗∗∗p< 0.0001 (ANOVA with Dunnett’s test). Also see Figure S1.

In search of long-lived mutants, we found that *xu120*, a mutant in our collection, showed increased lifespan (Figure 1A and Table S1). By whole-genome sequencing, we mapped the mutation to the *ears-2* gene that encodes the sole *C*. *elegans* mitochondrial glutamyl-tRNA synthetase. A single C to T transition in *xu120* leads to a predicted change of amino acid residue 152 from methionine to isoleucine (Figure 1B). To verify the phenotype, we inactivated *ears-2* by RNAi and found that RNAi of *ears-2* extended lifespan, similar to the phenotype of *ears-2(xu120)* mutant worms (Figure 1C and Table S1). Of interest, mutations in another mitochondrial ARS gene *lars-2/lrs-2*, also extend lifespan (Lee et al., 2003). These data together support the notion that inactivation of mitochondrial ARS genes prolongs longevity.

### Inactivation of *ears-2* alters mitochondrial function and activates UPR^mt^

*ears-2* encodes a mitochondrial ARS. Mitochondrial ARSs catalyze the loading of tRNA with cognate amino acids, which represents a key step in the translation of proteins encoded by the mitochondrial genome. In *C*. *elegans*, the mitochondrial genome encodes 12 proteins, all of which participate in the oxidative phosphorylation process essential for ATP production (Okimoto et al., 1992). We therefore examined whether inactivation of *ears-2* affects normal mitochondrial functions. We measured the ATP content and reactive oxygen species (ROS) level in *ears-2(RNAi)* worms, and found that RNAi of *ears-2* markedly reduced the ATP level while increasing the production of ROS (Figures 1D and 1E), indicating compromised mitochondrial functions.

In addition to compromising mitochondrial functions, interfering with mitochondrial mRNA translation is also known to trigger UPR^mt^, resulting in lifespan extension (Houtkooper et al., 2013). We thus wondered if inactivation of *ears-2* induces UPR^mt^. Using *hsp-6::GFP* as a UPR^mt^ reporter (Benedetti et al., 2006), we observed that *ears-2* RNAi induced UPR^mt^, similar to the effect of *cco-1* RNAi (Figures 1F and 1G), which served as a positive control (Durieux et al., 2011). The observed UPR^mt^ in *ears-2(RNAi)* worms is specific, since RNAi of *ears-2* did not activate UPR in the ER (UPR^ER^); nor did it stimulate heat shock response or DAF-16 signaling pathway (Figure S1). Taken together, these results demonstrate that inactivation of *ears-2* compromises mitochondrial functions and triggers UPR^mt^.

### *ears-2*-dependent lifespan extension requires UPR^mt^

**Figure 2:**
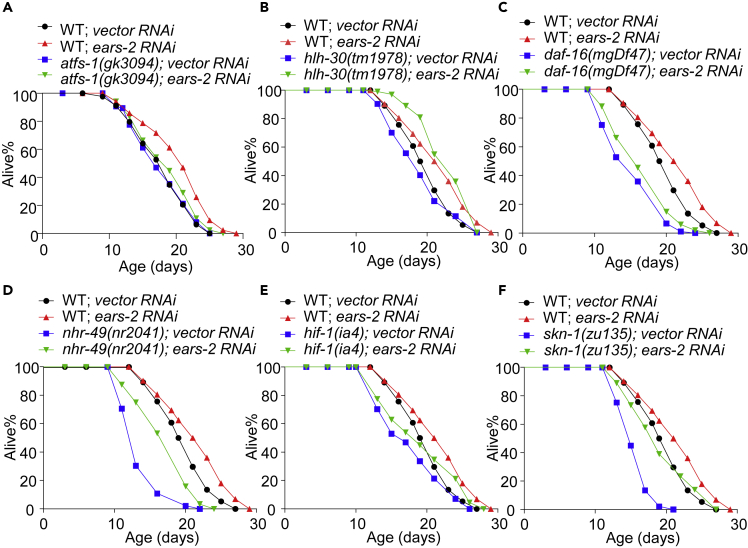
EARS-2-dependent lifespan regulation requires *atfs-1* (A) *atfs-1(gk3094)* mutation fully suppresses the long-lived phenotype of *ears-2* RNAi worms. (B–F) *hlh-30(tm1978)* (B), *daf-16(mgDf47)* (C), *nhr-49(nr2041)* (D), *hif-1(ia4)* (E) or *skn-1(zu135)* (F) mutation fails to fully suppress the long-lived phenotype of *ears-2* RNAi worms. B-F shared the same WT; *vector RNAi* curve and WT; *ears-2 RNAi* curve as they were done at the same time. All lifespan assays were performed at 20°C and repeated at least twice. See Table S1 for lifespan statistics. Logrank (Kaplan-Meier) was used to calculate p values.

We then asked if *ears-2*-dependent lifespan extension requires UPR^mt^. To address this question, we examined *atfs-1*, a key player in UPR^mt^ (Nargund et al., 2012), and found that mutations in *atfs-1* suppressed the lifespan-extending phenotype of *ears-2* RNAi worms (Figure 2A and Table S1). As a control, we also assayed mutants of several lifespan regulating transcription factors, such as *daf-16*, *nhr-49*, *hif-1*, *skn-1* and *hlh-30*, and found that they are not required for *ears-2*-dependent lifespan extension (Figures 2B–2F and Table S1). Notably, *ears-2(RNAi)* appears to suppress the short-lived phenotype of *hlh-30* mutant worms, suggesting a genetic interaction between the two genes (Figure 2B and Table S1). These data suggest that *ears-2*-dependent lifespan extension requires UPR^mt^, indicating an essential role of UPR^mt^ in mediating *ears-2* longevity.

### Inactivation of *ears-1* shortens lifespan

**Figure 3:**
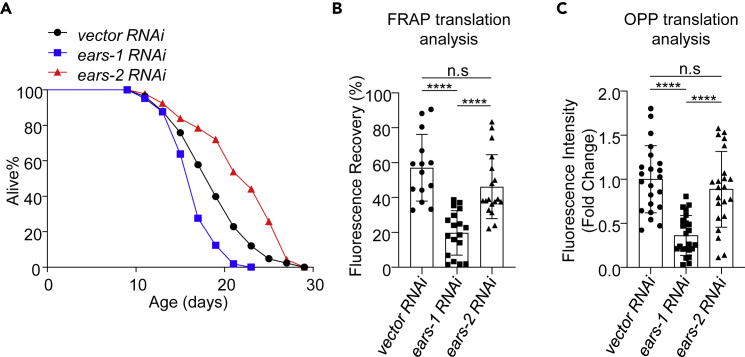
Inactivation of *ears-1* shortens lifespan and inhibits protein synthesis (A) *ears-1* RNAi shortens lifespan while *ears-2* RNAi extends lifespan. All lifespan assays were performed at 20°C and were repeated at least twice. See Table S1 for lifespan statistics. Logrank (Kaplan-Meier) was used to calculate p values. (B-C) *ears-1* RNAi inhibits protein translation. (B) FRAP translation analysis was used to quantify the rate of mCherry protein translation expressed as a transgene in the intestine under the *ges-1* promoter. n = 14–19. Error bars represent SE of mean. n.s. indicates no significant difference. ∗∗∗∗p< 0.0001 (ANOVA with Tukey’s test). (C) O-propargyl-puromycin (OPP) translation analysis was used to quantify the rate of total protein translation. n = 22–23. Error bars represent SE of mean. n.s. indicates no significant difference. ∗∗∗∗p< 0.0001 (ANOVA with Tukey’s test). Also see Figure S2.

While EARS-2 uploads glutamic acid to its cognate tRNA in the mitochondria, its cytoplasmic homolog EARS-1 does so in the cytoplasm. To evaluate whether inactivation of *ears-1* can also extend lifespan, we knocked down *ears-1* by RNAi. Surprisingly, RNAi of *ears-1* greatly shortened lifespan (Figure 3A and Table S1). This is rather surprising because it is well known that reducing translation extends lifespan. In the case of EARS-1, a key enzyme that produces charged tRNA^glu^ needed for the synthesis of all the proteins in the cytosol, its inactivation should presumably reduce the rate of translation and thereby extend lifespan. Indeed, using an FRAP-based assay to quantify the rate of translation of a fluorescent reporter protein expressed in the cytoplasm (Papandreou et al., 2020), or using the O-propargyl-puromycin (OPP) incorporation assay to quantify the translation rate of total proteins (Somers et al., 2022), we found that RNAi of *ears-1* indeed substantially inhibited the translation rate of the reporter protein (Figure 3B) or total proteins (Figure 3C). As a control, RNAi of *ears-2* only had a minimal effect (Figures 3B and 3C). We thus conclude that unlike *ears-2*, inactivation of its cytoplasmic homolog *ears-1* shortened lifespan, though such treatment indeed reduced the rate of translation.

### ARS genes encoded in the *C*. *elegans* genome

**Figure 4:**
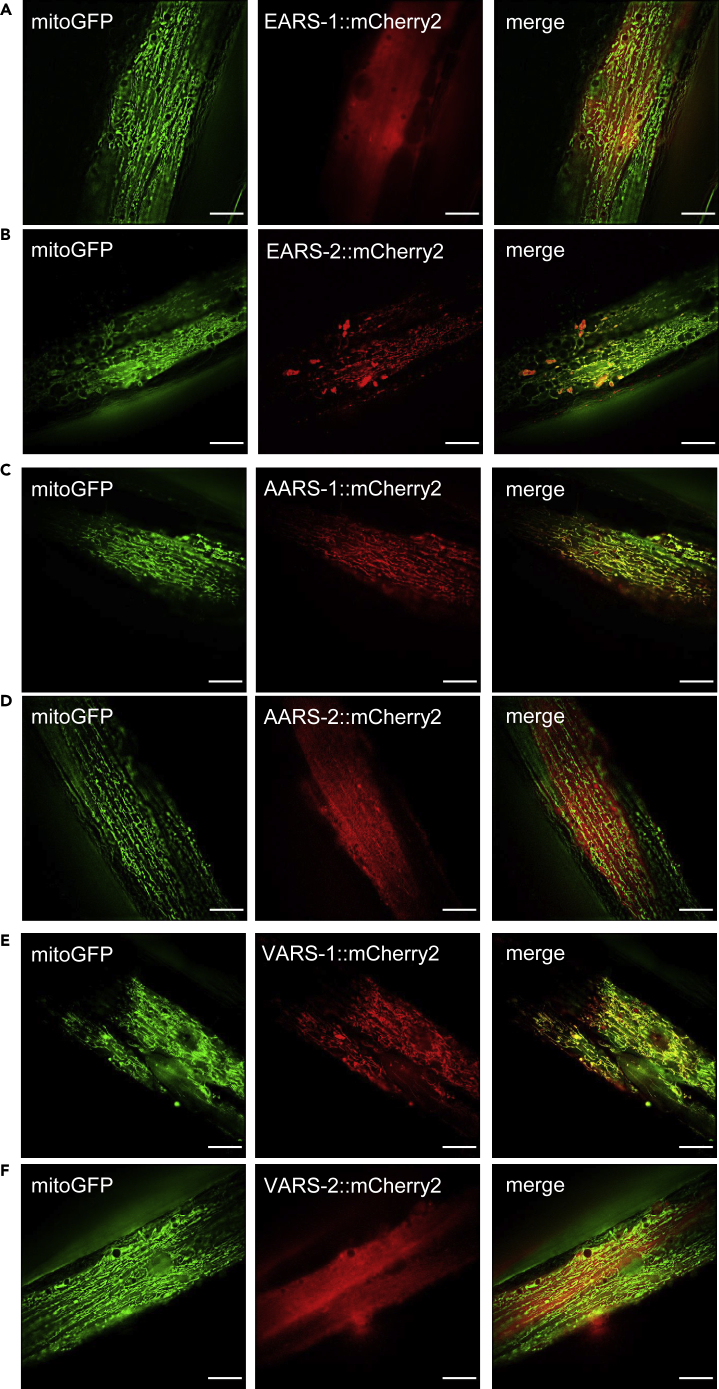
Subcellular localization of EARS-1, EARS-2, AARS-1, AARS-2, VARS-1 and VARS-2 (A–F) EARS-2::mCherry (B), AARS-1::mCherry (C) and VARS-1::mCherry (E) co-localize with mitoGFP, a mitochondria-targeted GFP marker, in body-wall muscle cells. EARS-1::mCherry (A), AARS-2::mCherry (D) and VARS-2::mCherry (F) do not co-localize with mitoGFP, but rather exhibit a more diffuse pattern in body-wall muscle cells. Transgenes were expressed in body wall muscle cells under the *myo-3* promoter, except *ears-2*::*mCherry* that was expressed under its own promoter. A commercial structured illumination microscope (HiS-SIM, High Sensitivity Structured Illumination Microscope) was used to acquire and reconstruct the images. Scale bars, 10 μm.

The observation that the mitochondria-specific ARS gene *ears-2* and the cytoplasm-specific ARS gene *ears-1* possess opposing roles in lifespan regulation prompted us to ask the question whether this is a phenomenon unique for glutamic acid-specific ARS genes like *ears-1* and *ears-2* or a general phenomenon for all ARS genes. We thus decided to systematically examine the role of all *C*. *elegans* ARS genes in longevity. As a first step to address this question, we surveyed the *C*. *elegans* genomic database (Wormbase) for ARS genes. There are 34 ARS genes encoded in the *C*. *elegans* genome, with 15 predicted to act exclusively in the cytoplasm, 13 in the mitochondria, and 6 in both the cytoplasm and mitochondria that are thus considered bifunctional (Table 1). These genes are named in a way similar to *ears-1* and *ears-2*, with cytoplasmic/bifunctional ARS members named class “*1*” genes and mitochondrial ARS members class “*2*” genes (Antonellis and Green, 2008). However, four genes do not follow this nomenclature, i.e. *aars-1*, *aars-2*, *vars-1*, and *vars-2*. Specifically, based on the sequence homology, *aars-1* and *vars-1* are predicted to be mitochondria-specific ARS genes, whereas *aars-2* and *vars-2* are expected to encode cytoplasm-specific ARSs (Table 1); yet their gene names are switched. To clarify this, we set out to determine the subcellular protein localization of these four ARSs by expressing them as an mCherry fusion protein in transgenic animals. We focused on body wall muscles, a cell type commonly used for visualizing mitochondrial morphology. We found that as expected, AARS-1::mCherry and VARS-1::mCherry co-localized with a mitochondrial GFP marker (mitoGFP), similar to the case with EARS-2::mCherry (Figure 4). By contrast, AARS-2::mCherry and VARS-2::mCherry did not, but rather exhibited a more diffuse pattern similar to EARS-1::mCherry (Figure 4). We thus conclude that as predicted by their gene sequences, *aars-1* and *vars-1* likely encode mitochondrial ARSs, while *aars-2* and *vars-2* may instead encode cytoplasmic ARSs.

**Table 1:** *C*. *elegans* aminoacyl-tRNA synthetases (ARSs)

Amino acid	Gene ID	Gene	Other names	Human ortholog	Predicted subcellular localization for *C. elegans* ARS	Identity
Arginine	F26F4.10	*rars-1*	*rrt-1*	*RARS1* (Cyto)	cytoplasm	46.379%
	C29H12.1	*rars-2*	*rrt-2*	*RARS2* (Mito)	mitochondrion	23.974%
Phenylalanine	T08B2.9	*fars-1*	*frs-1*, *let-396*	*FARSA* (Cyto)	cytoplasm	60.294%
	Y60A3A.13	*fars-2*	*frs-3*	*FARS2* (Mito)	mitochondrion	45.952%
	F22B5.9	*fars-3*	*frs-2*	*FARSB* (Cyto)	cytoplasm	59.290%
Alanine	W02B12.6	*aars-1*	*ars-1*, *alas*	*AARS2* (Mito)	mitochondrion	27.944%
	F28H1.3	*aars-2*	*ars-2*, *let-366*	*AARS1* (Cyto)	cytoplasm	56.18%
Leucine	R74.1	*lars-1*	*lrs-1*	*LARS1* (Cyto)	cytoplasm	56.334%
	ZK524.3	*lars-2*	*lrs-2*	*LARS2* (Mito)	mitochondrion	35.183%
Isoleucine	R11A8.6	*iars-1*	*irs-1*	*IARS1* (Cyto)	cytoplasm	56.083%
	C25A1.7	*iars-2*	*irs-2*	*IARS2* (Mito)	mitochondrion	33.730%
Tryptophane	Y80D3A.1	*wars-1*	*wrs-1*	*WARS1* (Cyto)	cytoplasm	61.965%
	C34E10.4	*wars-2*	*wrs-2*	*WARS2* (Mito)	mitochondrion	42.727%
Proline	T20H4.3	*pars-1*	*prs-1*	*EPRS1* (Cyto)	cytoplasm	53.909%
	T27F6.5	*pars-2*	*prs-2*	*PARS2* (Mito)	mitochondrion	93.305%
Valine	ZC513.4	*vars-1*	*vrs-1*	*VARS2* (Mito)	mitochondrion	31.267%
	Y87G2A.5	*vars-2*	*vrs-2*, *glp-4*	*VARS1* (Cyto)	cytoplasm	43.676%
Aspartic acid	B0464.1	*dars-1*	*drs-1*	*DARS1* (Cyto)	cytoplasm	63.786%
	F10C2.6	*dars-2*	*drs-2*	*DARS2* (Mito)	cytoplasm	42.105%
Glutamic acid	ZC434.5	*ears-1*	*ers-2*, *qrs-3*	*EPRS1* (Cyto)	cytoplasm	48.956%
	T07A9.2	*ears-2*	*ers-3*, *qrs-6*	*EARS2* (Mito)	mitochondrion	38.351%
Asparagine	F22D6.3	*nars-1*	*nrs-1*, *let-389*	*NARS1* (Cyto)	cytoplasm	59.259%
	Y66D12A.23	*nars-2*	*nrs-2*	*NARS2* (Mito)	ND	36.806%
Serine	C47E12.1	*sars-1*	*srs-2*	*SARS1* (Cyto)	cytoplasm	65.779%
	W03B1.4	*sars-2*	*srs-1*, *SerRS*	*SARS2* (Mito)	mitochondrion	41.587%
Tyrosine	Y105E8A.19	*yars-1*		*YARS1* (Cyto)	ND	57.143%
	K08F11.4	*yars-2*	*yrs-2*	*YARS2* (Mito)	cytoplasm/mitochondrion	29.448%
Histidine	T11G6.1	*hars-1*	*hrs-1*	*HARS1* (Cyto)	cytoplasm/mitochondrion	51.504%
				*HARS2* (Mito)		45.951%
Methionine	F58B3.5	*mars-1*	*mrs-1*, *let-65*	*MARS1*(Cyto)	cytoplasm/mitochondrion	32.087%
				*MARS2* (Mito)		14.478%
Cysteine	Y23H5A.7	*cars-1*	*crs-1*	*CARS1* (Cyto)	cytoplasm/mitochondrion	43.059%
	Y23H5A.1	*cars-2*	*pseudogene*			
				*CARS2* (Mito)		23.11%
Threonine	C47D12.6	*tars-1*	*trs-1*	*TARS1* (Cyto)	cytoplasm/mitochondrion	57.93%
				*TARS2* (Mito)		45.007%
Lysine	T02G5.9	*kars-1*	*krs-1*	*KARS1* (Cyto-Mito)	cytoplasm/mitochondrion	57.358%
Glycine	T10F2.1	*gars-1*	*grs-1*	*GARS1* (Cyto-Mito)	cytoplasm/mitochondrion	51.299%
Glutamine	Y41E3.4	*qars-1*	*ers-1*, *qrs-5*	*QARS1* (Cyto)	cytoplasm	54.488%

### Opposing roles of cytoplasmic and mitochondrial ARSs in lifespan regulation

**Figure 5:**
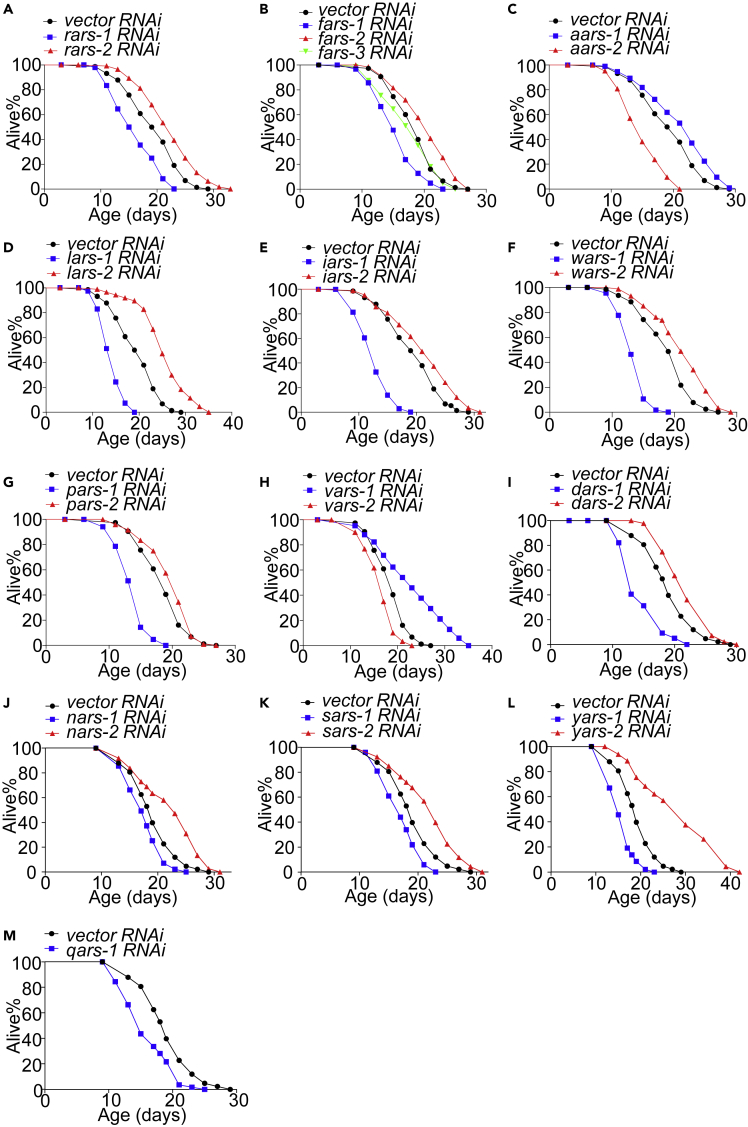
Inactivation of mitochondrial aminoacyl-tRNA synthetase genes extends lifespan, but inactivation of cytoplasmic aminoacyl-tRNA synthetase genes shortens lifespan (A–L) RNAi of mitochondrial arginyl tRNA synthetases gene *rars-2* (A), phenylalanyl tRNA synthetases gene *fars-2* (B), alanyl tRNA synthetases gene *aars-1* (C), leucinyl tRNA synthetases gene *lars-2* (D), isoleucinyl tRNA synthetases gene *iars-2* (E), tryptophanyl tRNA synthetases gene *wars-2* (F), prolyl tRNA synthetase gene *pars-2* (G), valinyl tRNA synthetase gene *vars-1* (H), aspartyl tRNA synthetase *dars-2* (I), asparaginyl tRNA synthetase gene *nars-2* (J), serinyl tRNA synthetase gene *sars-2* (K) and tyrosyl tRNA synthetase gene *yars-2* (L) extend lifespan. (A-M) RNAi of cytoplasmic arginyl tRNA synthetases gene *rars-1* (A), phenylalanyl tRNA synthetases gene *fars-1* (B), alanyl tRNA synthetases gene *aars-2* (C), leucinyl tRNA synthetases gene *lars-1* (D), isoleucinyl tRNA synthetases gene *iars-1* (E), tryptophanyl tRNA synthetases gene *wars-1* (F), prolyl tRNA synthetase gene *pars-1* (G), valinyl tRNA synthetase gene *vars-2* (H), aspartyl tRNA synthetase *dars-1* (I), asparaginyl tRNA synthetase gene *nars-1* (J), serinyl tRNA synthetase gene *sars-1* (K), tyrosyl tRNA synthetase gene *yars-1* (L) and glutaminyl tRNA synthetase gene *qars-1* (M) shorten lifespan. RNAi of cytoplasmic phenylalanyl tRNA synthetases gene *fars-3* (B) does not affect lifespan. A, C-E share the same vector RNAi curve as they were done at the same time. B, G and H share the same vector RNAi curve as they were done at the same time. I-L share the same *vector RNAi* curve as they were done at the same time. All lifespan assays were performed at 20°C and were repeated at least twice. See Table S1 for lifespan statistics. Logrank (Kaplan-Meier) was used to calculate p values.

**Figure 6:**
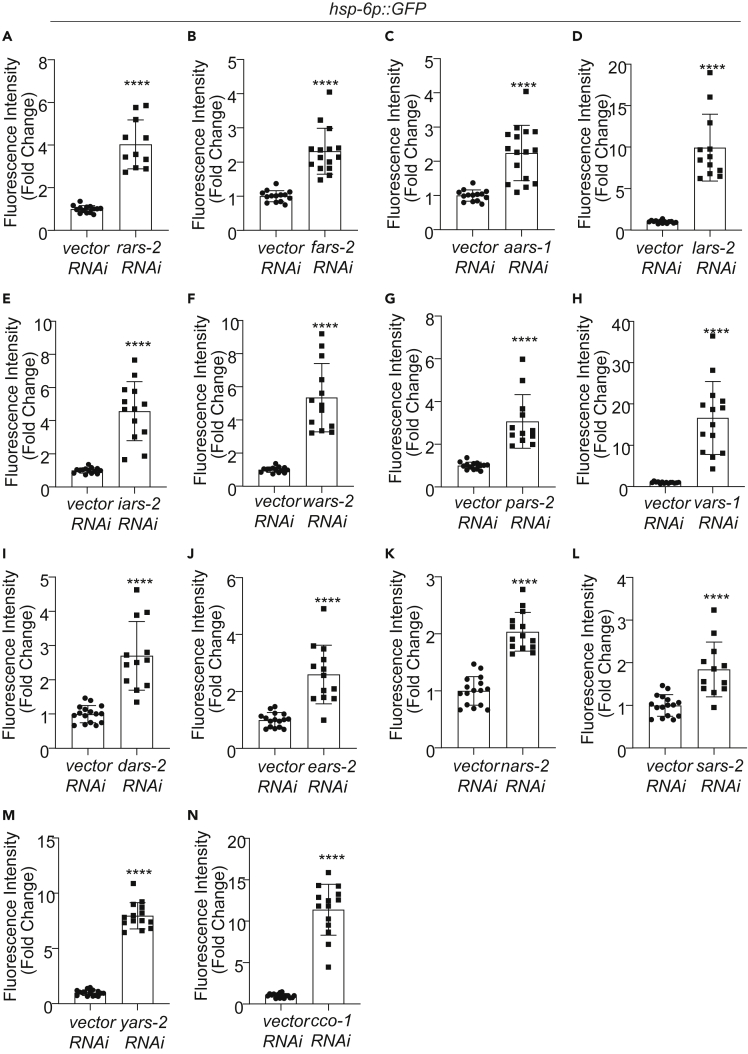
Inactivation of mitochondrial aminoacyl-tRNA synthetase genes activates UPR^mt^ (A–M) *RNAi* of all the mitochondrial aminoacyl-tRNA synthetase genes induces UPR^mt^. (A) *rars-2*, (B) *fars-2*, (C) *aars-1*, (D) *lars-2*, (E) *iars-2*, (F) *wars-2*, (G) *pars-2*, (H) *vars-1*, (I) *dars-2*, (J) *ears-2*, (K) *nars-2*, (L) *sars-2*, (M) *yars-2*, (N) *cco-1*. As a positive control, *cco-1* RNAi induced UPR^mt^. *hsp-6p:*:*GFP* transgene was used as a reporter for UPR^mt^. A–G share the same vector RNAi control as they were done at the same time. I-N share the same vector RNAi control as they were done at the same time. n = 11–16, Error bars represent SE of mean. ∗∗∗∗p< 0.0001 (t test). Also see Figures S3–S6.

We then inactivated all these 34 ARS genes by RNAi and assayed their lifespan. As was the case with *ears-2*, RNAi of all the 13 mitochondrial ARS genes extended lifespan (Figure 5). Similarly, inactivation of these mitochondrial ARS genes also induced UPR^mt^ (Figures 6 and S3), but not UPR^ER^ (Figure S4), heat shock response (Figure S5) or DAF-16 signaling pathway (Figure S6). By contrast, inactivation of all the 15 cytoplasmic ARS genes shortened lifespan, similar to the case with *ears-1* (Figure 5 and Table S1). RNAi of *nars-1* was previously reported to extend lifespan (Chen et al., 2007). We thus repeated this experiment using multiple conditions and observed a similar short-lived phenotype (Figures S7B–S7D). Thus, inactivation of cytoplasm- and mitochondria-specific ARS genes shortens and extends lifespan, respectively.

We performed RNAi on worms beginning at the L4 stage to avoid affecting growth and development. Indeed, we found that if initiated at the egg stage, RNAi knockdown of cytoplasmic-specific ARS genes caused lethality or larva arrest. As cytoplasmic ARSs mediate the translation of the vast majority of proteins in the cell, global shutdown of translation is expected to be detrimental to worms, thereby affecting their growth and development. By contrast, this phenomenon was not observed when we knocked down those 13 mitochondria-specific ARS genes. In fact, if we inactivated mitochondria-specific ARS genes beginning at the egg rather than L4 stage, the lifespan-extending effect from some of these genes, such as *pars-2*, was even more robust (Figure 5G vs. Figure S7A and Table S1). These results provide additional data supporting that inactivation of cytoplasm- and mitochondria-specific ARSs shortens and extends lifespan, respectively.

**Figure 7:**
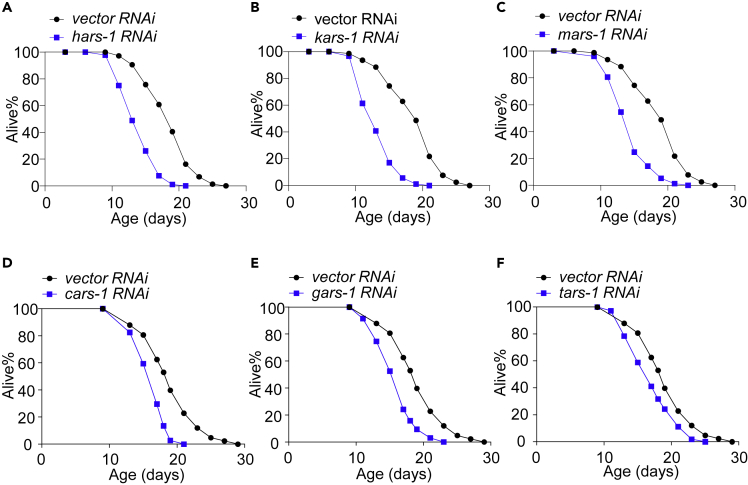
Inactivation of bifunctional aminoacyl-tRNA synthetase genes shortens lifespan (A–F) RNAi of all bifunctional aminoacyl-tRNA synthetase genes shortens lifespan. (A) histidyl tRNA synthetase gene *hars-1*, (B) lysyl tRNA synthetase gene *kars-1*, (C) methionyl tRNA synthetase gene *mars-1*, (D) cysteinyl tRNA synthetase gene *cars-1*, (E) glycyl tRNA synthetase gene *gars-1*, (F) threonyl tRNA synthetase gene *tars-1*. B and C share the same vector RNAi curve as they performed at the same time. D–F share the same vector RNAi curve as they were done at the same time. All lifespan assays were performed at 20°C and repeated at least twice. See Table S1 for lifespan statistics. Logrank (Kaplan-Meier) was used to calculate p values. Also see Figure S7.

Notably, inactivation of those 6 cyto-mito bifunctional ARS genes all shortened lifespan (Figure 7 and Table S1), similar to the case with *ears-1*. As RNAi of *tars-1* was previously reported to extend lifespan (Chen et al., 2007), we repeated this experiment under multiple conditions and observed a similar short-lived phenotype (Figures S7E–S7G). We thus conclude that inactivation of cyto-mito bifunctional ARS genes shortens lifespan. Because inhibiting the expression of these bifunctional ARS genes would reduce translation in both the cytoplasm and mitochondria, this suggests that the lifespan-shortening effect resulting from the inhibition of cytoplasmic translation predominates. This is expected, as nearly all the proteins in the cell are translated in the cytoplasm, whereas the mitochondria are merely in charge of the synthesis of 12 proteins. Together, these data demonstrate that reducing translation in the cytoplasm and mitochondria shortens and extends lifespan, respectively, revealing opposing roles of cytoplasmic and mitochondrial translation in lifespan regulation.

## Discussion

The notion that reducing translation can increase adult lifespan has been widely accepted, although the underlying mechanisms are largely unknown (Gonskikh and Polacek, 2017; Kennedy and Kaeberlein, 2009; Steffen and Dillin, 2016; Tavernarakis, 2008). Thus far, this view has been supported by experiments testing the major components in the translation apparatus, such as eIFs, eEFs, ribosomal proteins and ribosomal RNAs (Chen et al., 2007; Ching et al., 2010; Chiocchetti et al., 2007; Curran and Ruvkun, 2007; Essers et al., 2015; Hansen et al., 2007; Heissenberger et al., 2020; Houtkooper et al., 2013; Pan et al., 2007; Rogers et al., 2011; Schosserer et al., 2015; Syntichaki et al., 2007; Tiku et al., 2017; Xie et al., 2019). Nevertheless, whether this is the case for ARSs, a group of enzymes that play an essential role in translation, has not been extensively explored. In this study, we systematically assessed the role of all the ARS genes in longevity in *C*. *elegans*. We found that while inactivation of mitochondrial ARS genes extends lifespan and does so via UPR^mt^, inhibiting those ARSs acting in the cytoplasm, surprisingly, suppressed longevity, although such treatment indeed reduced the rate of translation. Thus, simply reducing the rate of translation may not extend lifespan.

While our observation that inactivation of mitochondrial ARS genes extends lifespan is consistent with the notion that reducing translation extends lifespan, our findings regarding cytoplasmic ARS genes are not. The question arises as to how to reconcile these findings with those demonstrating that reducing cytoplasmic translation extends lifespan. Notably, a previous study reported the intriguing observation that RNAi knockdown of the eukaryotic translation initiation factor 4G (eIF4G) results in a relative increase in the expression of stress response genes with long mRNA, although the overall rate of translation is reduced (Rogers et al., 2011). Knockdown of some of these stress response genes can suppress the long-lived phenotype induced by eIF4G inhibition, suggesting that a relatively increased expression of a select group of genes may underlie the longevity phenotype associated with eIF4G inhibition (Rogers et al., 2011). It is possible that inhibiting the expression or function of other eIFs, eEFs, and ribosomal proteins and RNAs may extend lifespan through a similar mechanism. One common feature of many such factors, such as eIFs and ribosomal proteins, is that they play a regulatory, rather than an essential role, in translation (Jackson et al., 2010; Wilson and Doudna Cate, 2012). For some other factors that are essential for translation such as eEF2 and ribosomal RNAs, the interventions that led to lifespan extension did not directly target such factors, but instead targeted their regulators that are likely not essential for translation (Heissenberger et al., 2020; Schosserer et al., 2015; Tiku et al., 2017; Xie et al., 2019). However, ARSs, particularly cytoplasmic ARSs, are enzymes essential for the synthesis of all the proteins in the cytoplasm, as each such ARS catalyzes the production of tRNA charged with a specific amino acid and thereby participates in the translation of nearly all the proteins in the cell (Kuo and Antonellis, 2020). Thus, direct inactivation of ARS genes would uniformly reduce the expression of all the proteins in the cytoplasm or mitochondria, making it less likely to induce a relatively increased expression of a select group of genes as shown for eIF4G. This may contribute to the short-lived phenotype associated with knockdown of cytoplasmic ARS genes.

By contrast, as the mitochondrial genome merely encodes 12 or 13 proteins, knockdown of mitochondria-specific ARSs would only affect a very small number of proteins. Thus, it is less likely for such knockdown to induce a severe detrimental effect on the cell; yet it can trigger UPR^mt^ that is known to promote longevity. We therefore suggest that the differential effects resulting from knockdown of cytoplasmic and mitochondrial ARS genes may underlie their opposing roles in lifespan regulation.

As an essential group of enzymes required for protein synthesis, ARSs are associated with a variety of human inherited diseases, ranging from peripheral neuropathies to infantile liver diseases and sensorineural hearing loss (Antonellis et al., 2003; Casey et al., 2012; Santos-Cortez et al., 2013). Of interest, mutations in different human ARS genes are linked to distinct clinical symptoms; yet they all are ubiquitously expressed in the same tissues/cells and all play a similar role in protein synthesis (Antonellis and Green, 2008; Kuo and Antonellis, 2020). The underlying mechanisms are not clear. Clearly, much remains to be learned about these enzymes. Our research in *C*. *elegans* points to a novel, complex role of ARSs in aging. Work in *C*. *elegans* has also revealed other roles of these enzymes in mediating stress responses such as resistance to hypoxia and starvation (Anderson et al., 2009; Webster et al., 2017). As ARSs genes are highly conserved from worms to mammals, research in *C*. *elegans* will continue to provide valuable insights into the function of ARS genes in health and disease.

### Limitations of the study

We show that RNAi knockdown of the cytoplasmic glutamyl-tRNA synthetase gene *ears-1* greatly shortens lifespan and substantially inhibits the translation rate of the reporter protein or total proteins. Nevertheless, we do not rule out the possibility that a slight reduction in the expression level of all cytosolic proteins might exhibit a beneficial effect, whereas too much of such reduction would be deleterious. To test this possibility, it would require more precise manipulation of the translation rate than that offered by the RNAi approach used in this study.

## STAR★Methods

### Key resources table

**Table:** 

REAGENT or RESOURCE	SOURCE	IDENTIFIER
**Bacterial and virus strains**		

*E*. *col*i: OP50	CGC	OP50
*E*. *col*i: OP50(xu363)	CGC	OP50

**Chemicals, peptides, and recombinant proteins**		

FUdR	Sigma	Cas# 50-91-9
Tetramisole	Sigma	Cas# 16595-80-5
Polystyrene beads solution	Polysciences	Cat# 00876-15
Carbenicillin	Sigma	Cat# 4800-94-6
isopropyl-β-D-thiogalactoside	Sigma	Cat# 367-93-1

Critical commercial assays		

ATP Assay Kit	Beyotime	Cat# S0026
Reactive Oxygen Species Assay Kit	Beyotime	Cat# S0033S
Click-iT™ Plus OPP Alexa Fluor™ 647	Invitrogen	Cat# C10458

**Experimental models: Organisms/strains**		

N2	CGC	N2
*ears-2(xu120) IV*	This paper	3574
*zcIs13[hsp-6p*::*GFP]*	CGC	SJ4100
*zcIs4[hsp-4p*::*GFP]*	CGC	AGD926
*pkIs1605[hsp-16*.*2p*::*GFP*::*LacZ + rol-6 (su1006)]*	CGC	NL3401
*muIs84[(pAD76) sod-3p*::*GFP + rol-6 (su1006)]*	CGC	CF1553
*atfs-1(gk3094) V*	CGC	VC3201
*hlh-30(tm1978) IV*	CGC	JIN1375
*daf-16(mgDf47) I*	Gary Ruvkun LAB	https://doi.org/10.1038/40194
*nhr-49(nr2041) I*	CGC	STE68
*hif-1(ia4) V*	CGC	ZG31
*skn-1(zu135) IV*	Bruce Bowerman LAB	https://doi.org/10.1016/0092-8674(9290078-q)
*zcIs17[ges-1p*::*mtGFP]*	CGC	SJ4143
*Ex411[ges-1p*::*SL2*::*mCherry2]*	This paper	3572

**Oligonucleotides**		

For information regarding oligonucleotide sequences used in this study please refer to Tables S2 and S3	This paper	Tables S2 and S3

**Recombinant DNA**		

Plasmid: L4440 vector RNAi	addgene	#1654
Plasmid: L4440 *ears-1* RNAi	This paper	p-527
Plasmid: L4440 *ears-2* RNAi	This paper	p-528
Plasmid: L4440 *hars-1* RNAi	This paper	p-529
Plasmid: L4440 *rars-1* RNAi	This paper	p-530
Plasmid: L4440 *rars-2* RNAi	This paper	p-531
Plasmid: L4440 *kars-1* RNAi	This paper	p-532
Plasmid: L4440 *fars-1* RNAi	This paper	p-533
Plasmid: L4440 *fars-2* RNAi	This paper	p-534
Plasmid: L4440 *fars-3* RNAi	This paper	p-535
Plasmid: L4440 *aars-1* RNAi	This paper	p-536
Plasmid: L4440 *aars-2* RNAi	This paper	p-537
Plasmid: L4440 *lars-1* RNAi	This paper	p-538
Plasmid: L4440 *lars-2* RNAi	This paper	p-539
Plasmid: L4440 *iars-1* RNAi	This paper	p-540
Plasmid: L4440 *iars-2* RNAi	This paper	p-541
Plasmid: L4440 *wars-1* RNAi	This paper	p-542
Plasmid: L4440 *wars-2* RNAi	Ahringer RNAi library	N/A
Plasmid: L4440 *pars-1* RNAi	This paper	p-543
Plasmid: L4440 *pars-2* RNAi	This paper	p-544
Plasmid: L4440 *vars-1* RNAi	This paper	p-545
Plasmid: L4440 *vars-2* RNAi	This paper	p-546
Plasmid: L4440 *dars-1* RNAi	This paper	p-547
Plasmid: L4440 *dars-2* RNAi	This paper	p-548
Plasmid: L4440 *nars-1* RNAi	This paper	p-549
Plasmid: L4440 *nars-2* RNAi	This paper	p-550
Plasmid: L4440 *sars-1* RNAi	This paper	p-551
Plasmid: L4440 *sars-2* RNAi	This paper	p-552
Plasmid: L4440 *yars-1* RNAi	This paper	p-553
Plasmid: L4440 *yars-2* RNAi	This paper	p-554
Plasmid: L4440 *ears-1* RNAi	This paper	p-555
Plasmid: L4440 *ears-2* RNAi	This paper	p-556
Plasmid: L4440 *mars-1* RNAi	This paper	p-557
Plasmid: L4440 *hars-1* RNAi	This paper	p-558
Plasmid: L4440 *kars-1* RNAi	This paper	p-559
Plasmid: L4440 *cars-1* RNAi	This paper	p-560
Plasmid: L4440 *gars-1* RNAi	This paper	p-561
Plasmid: L4440 *qars-1* RNAi	This paper	p-562
Plasmid: L4440 *tars-1* RNAi	This paper	p-563
Plasmid: *PBS77*::*ges-1p*::*SL2*::*mCherry2*	This paper	p-564
Plasmid: *PBS77*::*ears-2p*::*ears-2 (cDNA)*::*mCherry2*	This paper	p-565
Plasmid: *PBS77*::*myo-3p*:: *ears-1 (cDNA)*::*mCherry2*	This paper	p-566
Plasmid: *PBS77*::*myo-3p*:: *aars-1 (cDNA)*::*mCherry2*	This paper	p-567
Plasmid: *PBS77*::*myo-3p*:: *aars-2 (cDNA)*::*mCherry2*	This paper	p-568
Plasmid: *PBS77*::*myo-3p*:: *vars-1 (cDNA)*::*mCherry2*	This paper	p-569
Plasmid: *PBS77*::*myo-3p*:: *vars-2 (cDNA)*::*mCherry2*	This paper	p-570

**Software and algorithms**		

ImageJ	NIH	https://imagej.net/software/fiji/
GraphPad Prism	GraphPad	Version 8.0.2
SPSS Statistics	IBM	Version 21.0.0.0
MetaMorph	Molecular Devices Inc.	Version 7.8.0.0

### Resource availability

#### Lead contact

Further information and requests for resources and reagents should be directed to and will be fulfilled by the lead contact, X.Z. Shawn Xu (shawnxu@umich.edu).

#### Material availability

Plasmids and *C*. *elegans* strains used or generated in this study are listed in the key resources table, which will be available upon reasonable request.

### Experimental model and subject details

#### Animals

Worms were fed standard *E*. *coli* strain OP50 on NGM agar plates at 20°C. Mutants were verified using PCR-based genotyping and sequencing. All the strains used in this study are listed in the key resources table. Worms carrying extrachromosomal arrays were generated by injecting plasmid DNA into the hermaphrodite gonad. Mutant strains and integrated transgenic strains were outcrossed at least four times before use. All strains were kept as hermaphrodites without males. Day 1 adult worms were used for ATP quantification, ROS quantification and FRAP assay. Day 2 adult worms were used for OPP assay and HiS-SIM imaging. Day 3 adult worms were used for other microscopy assays.

### Method details

#### Worms synchronization

Strains were grown at 20°C for at least three generations before lifespan determination. Twenty day 2 adult worms were transferred to fresh 60-mm nematode growth medium (NGM) plates. The adult worms were removed from the plates after laying eggs for 4 h. The plates were placed at 20°C for two days. L4 larva were used to start the normal lifespan or RNAi assays.

#### Lifespan assay

Lifespan assays were performed on 60 mm NGM plates at 20°C as previously described (Chun et al., 2015). ∼120 worms of each genotype were used for lifespan assays and transferred every other day to fresh NGM plates. Survival rate was scored every 1–2 days. Worms were censored if they crawled off the plate, bagged, or exhibited protruding vulva. In all cases, the first day of adulthood was scored as day 1. Lifespan data was analyzed with GraphPad Prism 8 (GraphPad Software, Inc.) and IBM SPSS Statistics 21 (IBM, Inc.). Log-rank (Kaplan-Meier) was used to calculate p values.

#### RNA interference(RNAi) assay

RNAi was performed using the RNAi-compatible OP50 bacterial strain OP50(xu363) as previously described (Xiao et al., 2015). RNAi plates included carbenicillin (100 μg/mL, Sigma, Cas#4800-94-6) and isopropyl-β-D-thiogalactoside (1 mM, Sigma, Cas#367-93-1). OP50(xu363) bacteria with vector or RNAi plasmid were seeded on RNAi plates 2 days before experiment. Worms were fed RNAi bacteria from L4 stage. In a few cases, HT115 bacteria were used for RNAi with or without 5-Fluoro-2’-deoxyuridine (FUdR, Sigma, Cas#50-91-9) in those lifespan assays involving *nars-1* and *tars-1* genes in an effort to reproduce published results (Chen et al., 2007).

#### Quantification of ATP levels

Total ATP content was quantified with the ATP assay Kit by following manufacture’s instructions (Beyotime, S0026). ∼120 day 1 adult worms were used for each measurement. The luminescence was recorded with a MultiSkan SkyHigh Microplate Spectrophotometer (Thermo Fisher Scientific).

#### Quantification of ROS levels

Day 1 adult worms were incubated for 40 min in 10 μMol/L DCFH-DA (Beyotime, S0033S, diluted in M9 buffer), washed three times in M9 buffer, and mounted on 2% agarose pads for microscopy. Images were acquired on IX73 invert microscope (Olympus) under a 10X objective using MetaMorph (Molecular Devices Inc.) and analyzed with ImageJ (NIH).

#### Imaging analysis

To quantify *hsp-6p*::*GFP*, *hsp-16*.*2p*::*GFP*, *hsp-4p*::*GFP* and *sod-3p*::*GFP* fluorescence intensity, ∼15 day 3 adult worms were anesthetized with 10mM tetramisole (Sigma, Cas#16595-80-5) on 2% agarose pads. Images were acquired on IX73 invert microscope (Olympus) under 10X objectives with MetaMorph (Molecular Devices Inc.) and analyzed with ImageJ (NIH).

#### Fluorescence recovery after photobleaching (FRAP) assay

FRAP studies were performed as previously described (Kim et al., 2013; Papandreou et al., 2020). Day 1 adult worms were immobilized on 5–10% agarose pads with 10 μL polystyrene beads solution (Polysciences, 0.1μm, Cat# 00876-15). Next, the immobilized worms were placed under a 20 X objective on IX73 invert microscope (Olympus), and the reference fluorescent image was acquired before and after photobleaching. The samples were photobleached for 10 min until the fluorescent signal was quenched to 30-50% of the initial intensity. Worms were then individually recovered in NGM plates, and the fluorescent images were acquired 6 h after. Fluorescent images were analyzed with ImageJ (NIH). The fluorescence recovery ratio was calculated according to the formula:

FluorescenceRecovery(%)=(R−D)÷(O−D)

Where O, D and R represents the mean gray value of the images from unbleached, bleached and recovered worms, respectively.

#### O-propargyl-puromycin (OPP) translation assay

OPP translation assay was performed as previously described (Somers et al., 2022). RNAi bacteria were diluted to 2 × 10^9^ CFU/mL and incubated with 1% PFA for an hour. PFA was washed out with M9 buffer three times. PFA-killed bacteria were re-suspended to 2 × 10^8^ CFU/mL with NGM. ∼100 N2 worms were cultured on RNAi bacteria lawn from the L1 stage. Day 2 adult worms were incubated for 3 h at 20°C with gentle shaking in 2 × 10^8^ CFU/mL of PFA-killed bacteria with 10 μM OPP (Invitrogen, Cat# C10458). Worm pellets were washed three times with PBS. For fixation, 100 μL 4% PFA was added for 1 h at 10°C with shaking. After fixation, the worm pellet was washed three times with PBS. The fixed worms were incubated in 500 μL solution with 440 μL Click-iT reaction buffer, 2.5 μL of 500 mM Alexa Fluor picolyl azide 647, 10 μL 100 mM copper protectant, and 50 μL of the reaction buffer additive (Invitrogen, Cat# C10458). The worms were incubated overnight (16 h) at 10°C with shaking. The worm pellets were then washed three times in PBS with shaking for 30 min each time to remove unconjugated Alexa Fluor 647. Fluorescent images were acquired with a confocal laser scanning microscope (Olympus FV3000) and analyzed with ImageJ (NIH).

#### High sensitivity structured illumination microscopy (HiS-SIM)

*zcIs14[myo-3p*::*mtGFP]* were micro-injected with *PBS77*::*myo-3p*::*ears-1(cDNA)*::*mCherry2*, *PBS77*::*ears-2p*::*ears-2(cDNA)*::*mCherry2*, *PBS77*::*myo-3p*::*aars-1(cDNA)*::*mCherry2*, *PBS77*::*myo-3p*::*aars-2(cDNA)*::*mCherry2*, *PBS77*::*myo-3p*::*vars-1(cDNA)*::*mCherry2*, *PBS77*::*myo-3p*::*vars-2(cDNA)*::*mCherry2*, *PBS77*::*myo-3p*::*hars-1(cDNA)*::*mCherry2* plasmids, respectively. Primers for cDNAs cloning are shown in Table S3.

Progeny (F1) of injected parents was directly used for imaging analysis. Briefly, day 2 adult F1 worms expressing mCherry were immobilized with 10 mM tetramisole (Sigma, Cas#16595-80-5) and mounted on 2% agarose pads. The procedure for Hessian imaging was performed as previously described (Huang et al., 2018). A commercial structured illumination microscope (HiS-SIM) was used to acquire and reconstruct the worm images. To further improve the resolution and contrast in reconstructed images, sparse deconvolution was used as previously described (Zhao et al., 2021). Images were further processed with software ImageJ (NIH).

### Quantification and statistical analysis

For the lifespan assays, survival graphs were generated using Prism 8 (GraphPad Software, Inc.) and IBM SPSS Statistics 21 (IBM, Inc.) software. Log-rank (Kaplan-Meier) was used to calculate p values. ImageJ Fiji (NIH) was used to analyze fluorescent images. The statistical analysis was performed in GraphPad Prism 8 (GraphPad Software, Inc.) software using t-test, ANOVA with Tukey’s test or ANOVA with Dunnett’s test to calculate p values. p values less than 0.05 were considered statistically significant.

## Data Availability

• All data reported in this paper will be shared by the lead contact upon request. • This paper did not generate original code. • Any additional information required to reanalyze the data reported in this paper is available from the lead contact upon request. • The datasets generated and analyzed during this study are either included within the manuscript or are available from the authors upon request.

## Supplemental information

Document S1. Figures S1–S7 and Tables S2 and S3

Table S1. Summary of lifespan results, related to Figures 1–3, 5, and 7

## References

[bib1] Anderson L.L., Mao X., Scott B.A., Crowder C.M. (2009). Survival from hypoxia in C. elegans by inactivation of aminoacyl-tRNA synthetases. Science.

[bib2] Antonellis A., Ellsworth R.E., Sambuughin N., Puls I., Abel A., Lee-Lin S.Q., Jordanova A., Kremensky I., Christodoulou K., Middleton L.T. (2003). Glycyl tRNA synthetase mutations in Charcot-Marie-Tooth disease type 2D and distal spinal muscular atrophy type V. Am. J. Hum. Genet..

[bib3] Antonellis A., Green E.D. (2008). The role of aminoacyl-tRNA synthetases in genetic diseases. Annu. Rev. Genomics Hum. Genet..

[bib4] Benedetti C., Haynes C.M., Yang Y., Harding H.P., Ron D. (2006). Ubiquitin-like protein 5 positively regulates chaperone gene expression in the mitochondrial unfolded protein response. Genetics.

[bib5] Casey J.P., McGettigan P., Lynam-Lennon N., McDermott M., Regan R., Conroy J., Bourke B., O'Sullivan J., Crushell E., Lynch S., Ennis S. (2012). Identification of a mutation in LARS as a novel cause of infantile hepatopathy. Mol. Genet. Metab..

[bib6] Casey J.P., Slattery S., Cotter M., Monavari A.A., Knerr I., Hughes J., Treacy E.P., Devaney D., McDermott M., Laffan E. (2015). Clinical and genetic characterisation of infantile liver failure syndrome type 1, due to recessive mutations in LARS. J. Inherit. Metab. Dis..

[bib7] Chen D., Pan K.Z., Palter J.E., Kapahi P. (2007). Longevity determined by developmental arrest genes in Caenorhabditis elegans. Aging Cell.

[bib8] Chihara T., Luginbuhl D., Luo L. (2007). Cytoplasmic and mitochondrial protein translation in axonal and dendritic terminal arborization. Nat. Neurosci..

[bib9] Ching T.T., Paal A.B., Mehta A., Zhong L., Hsu A.L. (2010). drr-2 encodes an eIF4H that acts downstream of TOR in diet-restriction-induced longevity of C. elegans. Aging Cell.

[bib10] Chiocchetti A., Zhou J., Zhu H., Karl T., Haubenreisser O., Rinnerthaler M., Heeren G., Oender K., Bauer J., Hintner H. (2007). Ribosomal proteins Rpl10 and Rps6 are potent regulators of yeast replicative life span. Exp. Gerontol..

[bib11] Chun L., Gong J., Yuan F., Zhang B., Liu H., Zheng T., Yu T., Xu X.Z.S., Liu J. (2015). Metabotropic GABA signalling modulates longevity in C. elegans. Nat. Commun..

[bib12] Curran S.P., Ruvkun G. (2007). Lifespan regulation by evolutionarily conserved genes essential for viability. PLoS Genet..

[bib13] Demontis F., Perrimon N. (2010). FOXO/4E-BP signaling in Drosophila muscles regulates organism-wide proteostasis during aging. Cell.

[bib14] Durieux J., Wolff S., Dillin A. (2011). The cell-non-autonomous nature of electron transport chain-mediated longevity. Cell.

[bib15] Essers P.B., Nonnekens J., Goos Y.J., Betist M.C., Viester M.D., Mossink B., Lansu N., Korswagen H.C., Jelier R., Brenkman A.B., MacInnes A.W. (2015). A long noncoding RNA on the ribosome is required for lifespan extension. Cell Rep..

[bib16] Friederich M.W., Timal S., Powell C.A., Dallabona C., Kurolap A., Palacios-Zambrano S., Bratkovic D., Derks T.G.J., Bick D., Bouman K. (2018). Pathogenic variants in glutamyl-tRNA(Gln) amidotransferase subunits cause a lethal mitochondrial cardiomyopathy disorder. Nat. Commun..

[bib17] Gonskikh Y., Polacek N. (2017). Alterations of the translation apparatus during aging and stress response. Mech. Ageing Dev..

[bib18] Hansen M., Taubert S., Crawford D., Libina N., Lee S.J., Kenyon C. (2007). Lifespan extension by conditions that inhibit translation in Caenorhabditis elegans. Aging Cell.

[bib19] Heissenberger C., Rollins J.A., Krammer T.L., Nagelreiter F., Stocker I., Wacheul L., Shpylovyi A., Tav K., Snow S., Grillari J. (2020). The ribosomal RNA m(5)C methyltransferase NSUN-1 modulates healthspan and oogenesis in Caenorhabditis elegans. Elife.

[bib20] Houtkooper R.H., Mouchiroud L., Ryu D., Moullan N., Katsyuba E., Knott G., Williams R.W., Auwerx J. (2013). Mitonuclear protein imbalance as a conserved longevity mechanism. Nature.

[bib21] Hu Z., Xia B., Postnikoff S.D., Shen Z.J., Tomoiaga A.S., Harkness T.A., Seol J.H., Li W., Chen K., Tyler J.K. (2018). Ssd1 and Gcn2 suppress global translation efficiency in replicatively aged yeast while their activation extends lifespan. Elife.

[bib22] Huang X., Fan J., Li L., Liu H., Wu R., Wu Y., Wei L., Mao H., Lal A., Xi P. (2018). Fast, long-term, super-resolution imaging with Hessian structured illumination microscopy. Nat. Biotechnol..

[bib23] Jackson R.J., Hellen C.U.T., Pestova T.V. (2010). The mechanism of eukaryotic translation initiation and principles of its regulation. Nat. Rev. Mol. Cell Biol..

[bib24] Jordanova A., Irobi J., Thomas F.P., Van Dijck P., Meerschaert K., Dewil M., Dierick I., Jacobs A., De Vriendt E., Guergueltcheva V. (2006). Disrupted function and axonal distribution of mutant tyrosyl-tRNA synthetase in dominant intermediate Charcot-Marie-Tooth neuropathy. Nat. Genet..

[bib25] Kennedy B.K., Kaeberlein M. (2009). Hot topics in aging research: protein translation, 2009. Aging Cell.

[bib26] Kenyon C.J. (2010). The genetics of ageing. Nature.

[bib27] Kim E., Sun L., Gabel C.V., Fang-Yen C. (2013). Long-term imaging of Caenorhabditis elegans using nanoparticle-mediated immobilization. PLoS One.

[bib28] Kuo M.E., Antonellis A. (2020). Ubiquitously expressed proteins and restricted phenotypes: exploring cell-specific sensitivities to impaired tRNA charging. Trends Genet..

[bib29] Kuo M.E., Theil A.F., Kievit A., Malicdan M.C., Introne W.J., Christian T., Verheijen F.W., Smith D.E.C., Mendes M.I., Hussaarts-Odijk L. (2019). Cysteinyl-tRNA synthetase mutations cause a multi-system, recessive disease that includes microcephaly, developmental delay, and brittle hair and nails. Am. J. Hum. Genet..

[bib30] Latour P., Thauvin-Robinet C., Baudelet-Mery C., Soichot P., Cusin V., Faivre L., Locatelli M.C., Mayencon M., Sarcey A., Broussolle E. (2010). A major determinant for binding and aminoacylation of tRNA(Ala) in cytoplasmic Alanyl-tRNA synthetase is mutated in dominant axonal Charcot-Marie-Tooth disease. Am. J. Hum. Genet..

[bib31] Lee S.S., Lee R.Y., Fraser A.G., Kamath R.S., Ahringer J., Ruvkun G. (2003). A systematic RNAi screen identifies a critical role for mitochondria in C. elegans longevity. Nat. Genet..

[bib32] Mishra P., Chan D.C. (2014). Mitochondrial dynamics and inheritance during cell division, development and disease. Nat. Rev. Mol. Cell Biol..

[bib33] Molenaars M., Janssens G.E., Williams E.G., Jongejan A., Lan J., Rabot S., Joly F., Moerland P.D., Schomakers B.V., Lezzerini M. (2020). A conserved mito-cytosolic translational balance links two longevity pathways. Cell Metab..

[bib34] Nagao A., Suzuki T., Katoh T., Sakaguchi Y., Suzuki T. (2009). Biogenesis of glutaminyl-mt tRNAGln in human mitochondria. Proc. Natl. Acad. Sci. USA.

[bib35] Nargund A.M., Pellegrino M.W., Fiorese C.J., Baker B.M., Haynes C.M. (2012). Mitochondrial import efficiency of ATFS-1 regulates mitochondrial UPR activation. Science.

[bib36] Okimoto R., Macfarlane J.L., Clary D.O., Wolstenholme D.R. (1992). The mitochondrial genomes of two nematodes, Caenorhabditis elegans and Ascaris suum.. Genetics.

[bib37] Pan K.Z., Palter J.E., Rogers A.N., Olsen A., Chen D., Lithgow G.J., Kapahi P. (2007). Inhibition of mRNA translation extends lifespan in Caenorhabditis elegans. Aging Cell.

[bib38] Papandreou M.E., Palikaras K., Tavernarakis N. (2020). Assessment of de novo Protein Synthesis Rates in Caenorhabditis elegans. J. Vis. Exp..

[bib39] Peroutka C., Salas J., Britton J., Bishop J., Kratz L., Gilmore M.M., Fahrner J.A., Golden W.C., Wang T. (2018). Severe neonatal manifestations of infantile liver failure syndrome type 1 caused by cytosolic leucine-tRNA synthetase deficiency. JIMD Rep..

[bib40] Rogers A.N., Chen D., McColl G., Czerwieniec G., Felkey K., Gibson B.W., Hubbard A., Melov S., Lithgow G.J., Kapahi P. (2011). Life span extension via eIF4G inhibition is mediated by posttranscriptional remodeling of stress response gene expression in C. elegans. Cell Metab..

[bib41] Santos-Cortez R.L., Lee K., Azeem Z., Antonellis P.J., Pollock L.M., Khan S., Irfanullah, Andrade-Elizondo P.B., Chiu I., Adams M.D., Basit S. (2013). Mutations in KARS, encoding lysyl-tRNA synthetase, cause autosomal-recessive nonsyndromic hearing impairment DFNB89. Am. J. Hum. Genet..

[bib42] Schosserer M., Minois N., Angerer T.B., Amring M., Dellago H., Harreither E., Calle-Perez A., Pircher A., Gerstl M.P., Pfeifenberger S. (2015). Methylation of ribosomal RNA by NSUN5 is a conserved mechanism modulating organismal lifespan. Nat. Commun..

[bib43] Somers H.M., Fuqua J.H., Bonnet F.X.A., Rollins J.A. (2022). Quantification of tissue-specific protein translation in whole C. elegans using O-propargyl-puromycin labeling and fluorescence microscopy. Cell Rep. Methods.

[bib44] Sonenberg N., Hinnebusch A.G. (2007). New modes of translational control in development, behavior, and disease. Mol. Cell.

[bib45] Steffen K.K., Dillin A. (2016). A ribosomal perspective on proteostasis and aging. Cell Metab..

[bib46] Steffen K.K., MacKay V.L., Kerr E.O., Tsuchiya M., Hu D., Fox L.A., Dang N., Johnston E.D., Oakes J.A., Tchao B.N. (2008). Yeast life span extension by depletion of 60s ribosomal subunits is mediated by Gcn4. Cell.

[bib47] Syntichaki P., Troulinaki K., Tavernarakis N. (2007). eIF4E function in somatic cells modulates ageing in Caenorhabditis elegans. Nature.

[bib48] Tavernarakis N. (2008). Ageing and the regulation of protein synthesis: a balancing act?. Trends Cell Biol..

[bib49] Theil A.F., Botta E., Raams A., Smith D.E.C., Mendes M.I., Caligiuri G., Giachetti S., Bione S., Carriero R., Liberi G. (2019). Bi-Allelic TARS mutations are associated with brittle hair phenotype. Am. J. Hum. Genet..

[bib50] Tiku V., Jain C., Raz Y., Nakamura S., Heestand B., Liu W., Spath M., Suchiman H.E.D., Muller R.U., Slagboom P.E. (2017). Small nucleoli are a cellular hallmark of longevity. Nat. Commun..

[bib51] Tolkunova E., Park H., Xia J., King M.P., Davidson E. (2000). The human lysyl-tRNA synthetase gene encodes both the cytoplasmic and mitochondrial enzymes by means of an unusual alternative splicing of the primary transcript. J. Biol. Chem..

[bib52] Tsai P.C., Soong B.W., Mademan I., Huang Y.H., Liu C.R., Hsiao C.T., Wu H.T., Liu T.T., Liu Y.T., Tseng Y.T. (2017). A recurrent WARS mutation is a novel cause of autosomal dominant distal hereditary motor neuropathy. Brain.

[bib53] Vester A., Velez-Ruiz G., McLaughlin H.M., Program N.C.S., Lupski J.R., Talbot K., Vance J.M., Zuchner S., Roda R.H., Fischbeck K.H. (2013). A loss-of-function variant in the human histidyl-tRNA synthetase (HARS) gene is neurotoxic in vivo. Hum. Mutat..

[bib54] Webster C.M., Pino E.C., Carr C.E., Wu L., Zhou B., Cedillo L., Kacergis M.C., Curran S.P., Soukas A.A. (2017). Genome-wide RNAi screen for fat regulatory genes in C. elegans identifies a proteostasis-AMPK Axis critical for starvation survival. Cell Rep..

[bib55] Wilson D.N., Doudna Cate J.H. (2012). The structure and function of the eukaryotic ribosome. Cold Spring Harb. Perspect. Biol..

[bib56] Xiao R., Chun L., Ronan E.A., Friedman D.I., Liu J., Xu X.Z. (2015). RNAi interrogation of dietary modulation of development, metabolism, behavior, and aging in C. elegans. Cell Rep..

[bib57] Xie J., de Souza Alves V., von der Haar T., O'Keefe L., Lenchine R.V., Jensen K.B., Liu R., Coldwell M.J., Wang X., Proud C.G. (2019). Regulation of the elongation phase of protein synthesis enhances translation accuracy and modulates lifespan. Curr. Biol..

[bib58] Zhao W., Zhao S., Li L., Huang X., Xing S., Zhang Y., Qiu G., Han Z., Shang Y., Sun D.E. (2021). Sparse deconvolution improves the resolution of live-cell super-resolution fluorescence microscopy. Nat. Biotechnol..

[bib59] Zhou X.L., He L.X., Yu L.J., Wang Y., Wang X.J., Wang E.D., Yang T. (2017). Mutations in KARS cause early-onset hearing loss and leukoencephalopathy: potential pathogenic mechanism. Hum. Mutat..

