# Supplementary material for: Cytoplasmic and mitochondrial aminoacyl-tRNA synthetases differentially regulate lifespan in Caenorhabditis elegans
Source: iScience. 2022 Oct 3;25(11):105266. doi: 10.1016/j.isci.2022.105266 (PMC9593246; doi:10.1016/j.isci.2022.105266)
Supplement: Document S1. Figures S1–S7 and Tables S2 and S3 [file mmc1.pdf]

iScience, Volume *25*

Supplemental information

Cytoplasmic and mitochondrial aminoacyl-tRNA synthetases differentially regulate lifespan in *Caenorhabditis elegans*

Tianlin Zheng, Qiang Luo, Chengxuan Han, Jiejun Zhou, Jianke Gong, Lei Chun, X.Z. Shawn Xu, and Jianfeng Liu

Figure S1

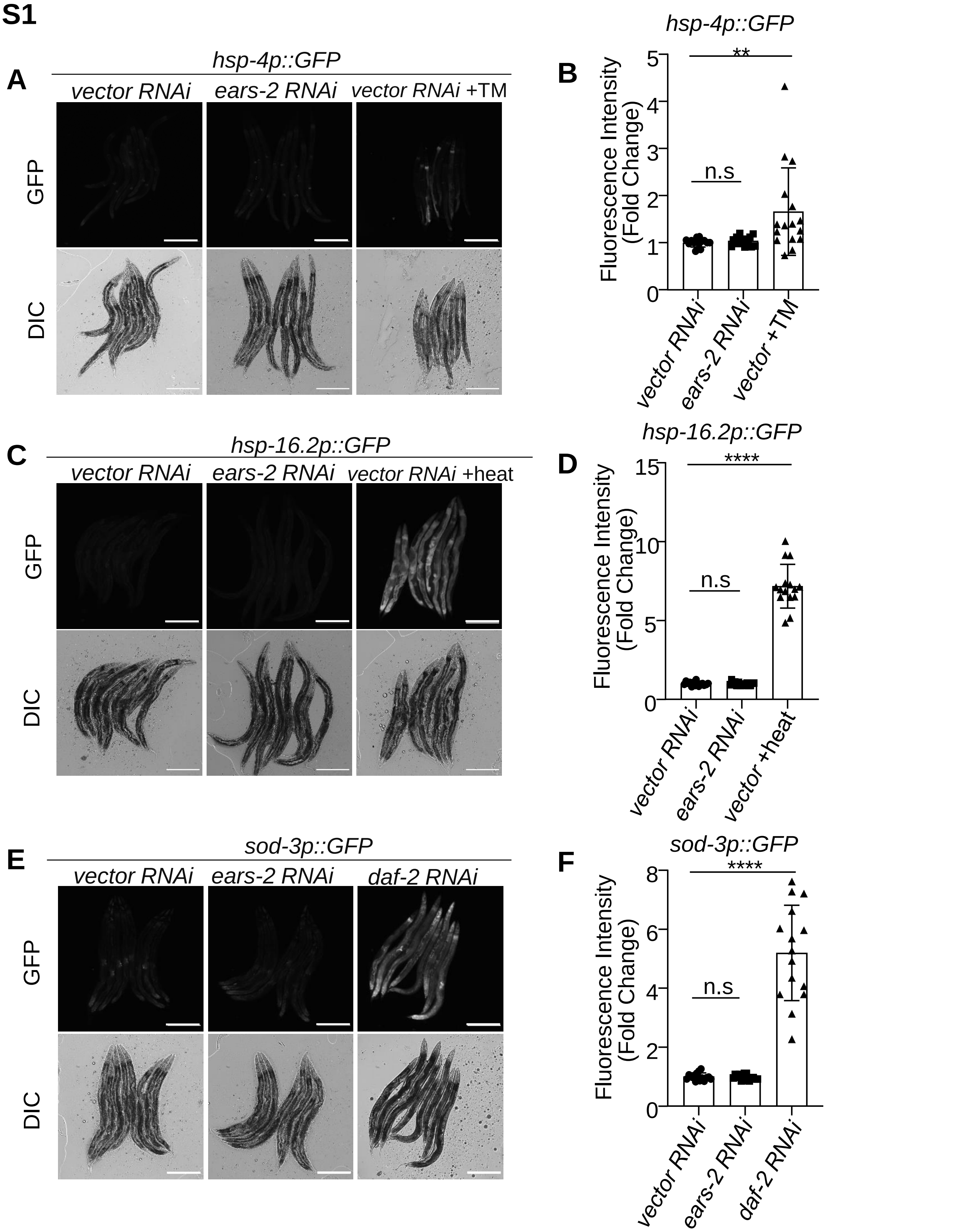

Figure S1. *ears-2* RNAi does not activate UPR^{ER}, heat shock response or DAF-16 pathway. Related to Figure 1.

(A-B) *ears-2* RNAi does not induce UPR^{ER}. As a positive control, UPR^{ER} can be induced in *hsp-4p::GFP* transgenic reporter worms upon treatment with tunicamycin (TM) for 2 hr. Representative images (A) and quantification graph (B) are shown.

(C-D) *ears-2* RNAi does not induce heat shock response. As a positive control, heat shock (1 hr at 33 °C) induced heat shock response in *hsp-16.2p::GFP* transgenic reporter worms. Representative images (C) and quantification graph (D) are shown.

(E-F) *ears-2* RNAi does not activate DAF-16 signaling pathway. As a positive control, *daf-2* RNAi upregulated the expression level of *sod-3p::GFP*, a commonly used reporter for DAF-16 activity. Representative images (E) and quantification graph (F) are shown. Scale bars, 300 μm. n ≥15. Error bars represent standard error of mean. n.s. indicates no significant difference. ***P*<0.01, *****P*<0.0001 (ANOVA with Dunnett's test).

Figure S2

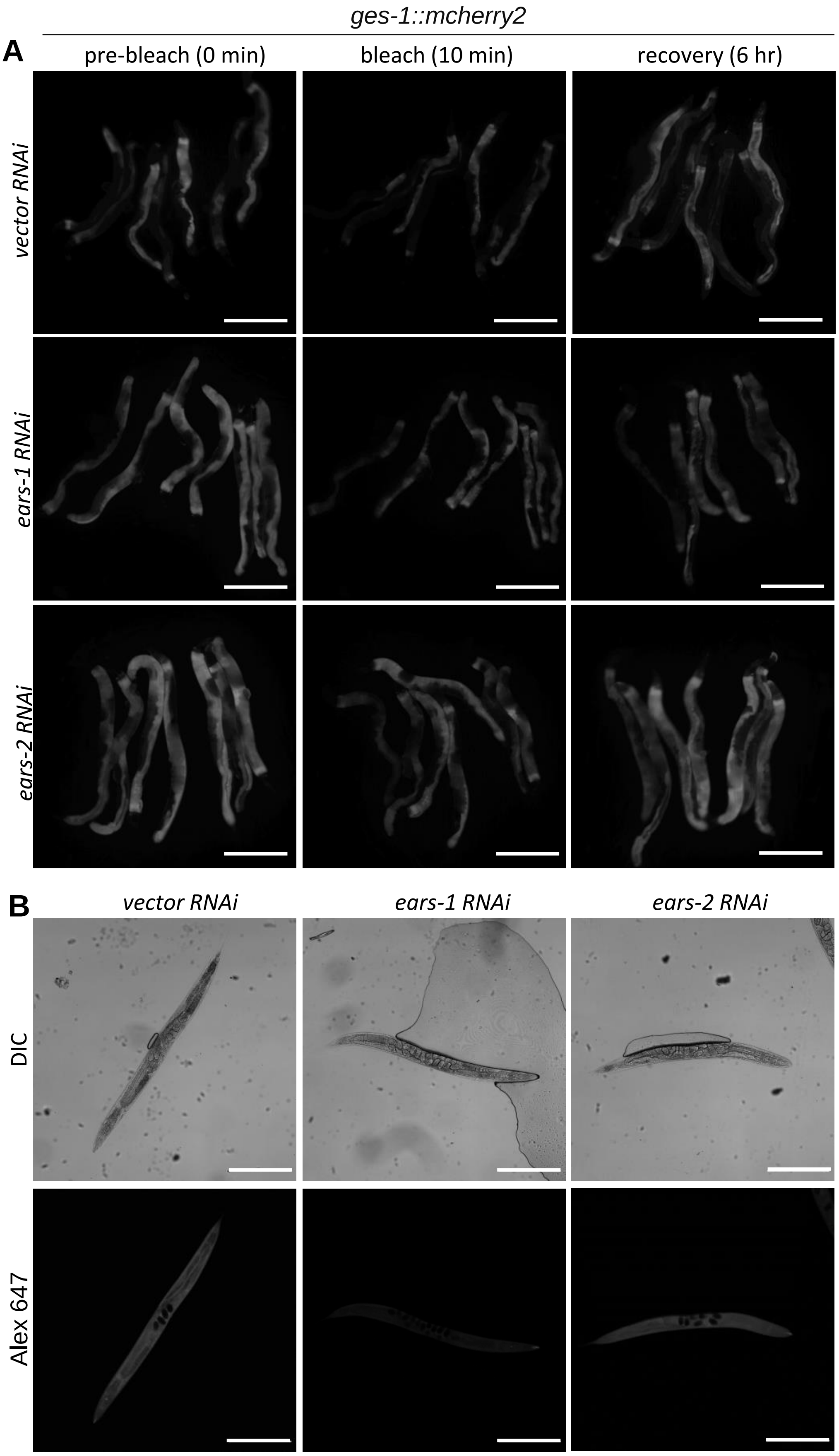

Figure S2 *ears-1* RNAi inhibits protein translation. Related to Figure 3.
(A) FRAP translation analysis. Representative images for Figure 3B. All worms carry a *ges-1::mCherry* transgene. Scale bars, 300 μm.
(B) O-propargyl-puromycin (OPP) translation analysis. Representative images for Figure 3C. Scale bars, 300 μm.

Figure S3

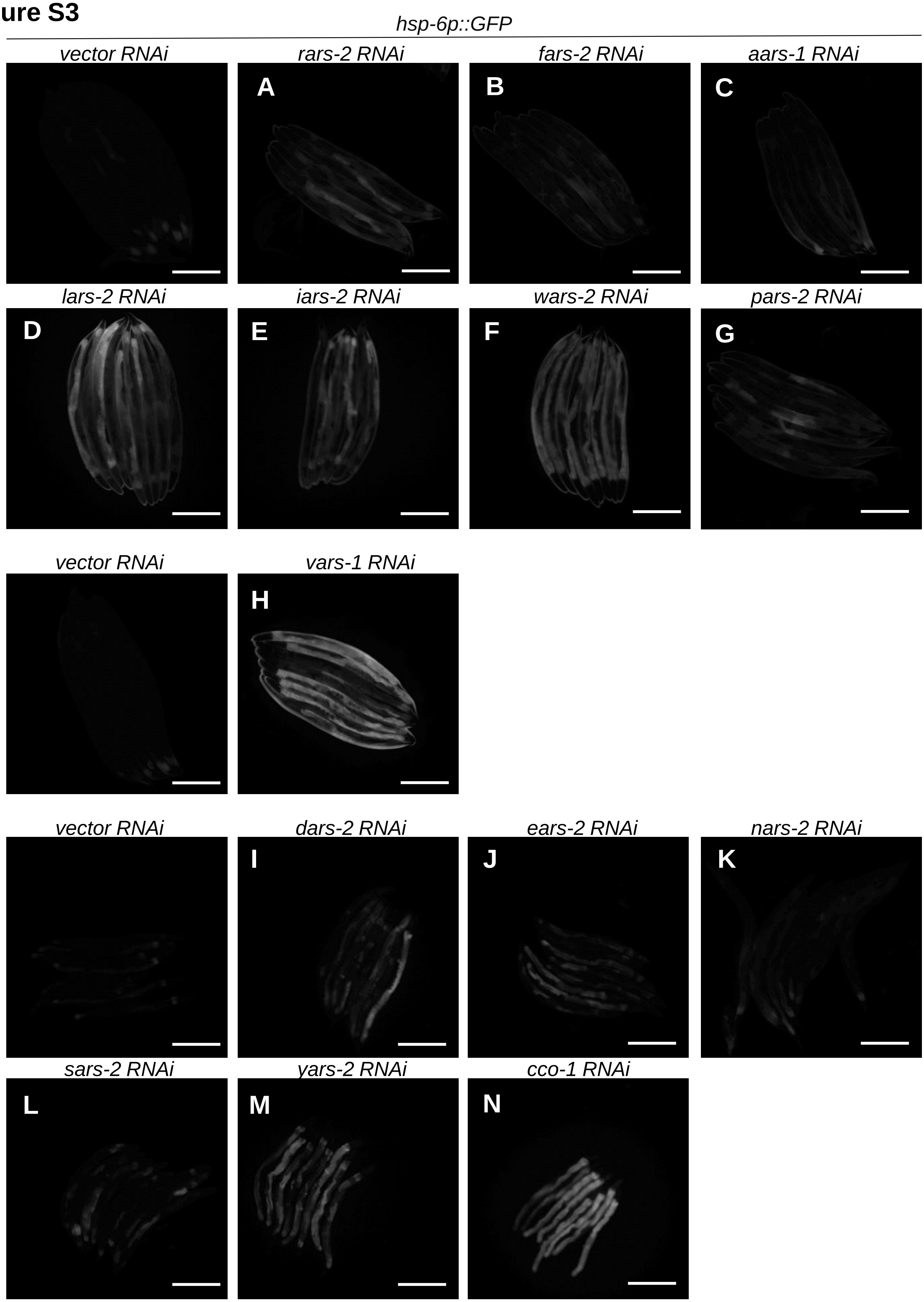

Figure S3 Representative images for Figure 6. Related to Figure 6.
All worms carry a *hsp-6p::GFP* transgenic UPR^{mt} reporter. (A) *rars-2 RNAi*, (B) *fars-2 RNAi*, (C) *aars-1 RNAi*, (D) *lars-2 RNAi*, (E) *iars-2 RNAi*, (F) *wars-2 RNAi*, (G) *pars-2 RNAi*, (H) *vars-1 RNAi*, (I) *dars-2 RNAi*, (J) *ears-2 RNAi*, (K) *nars-2 RNAi*, (L) *sars-2 RNAi*, (M) *yars-2 RNAi*, (N) *cco-1 RNAi*. Scale bars, 300 μm.

Figure S4

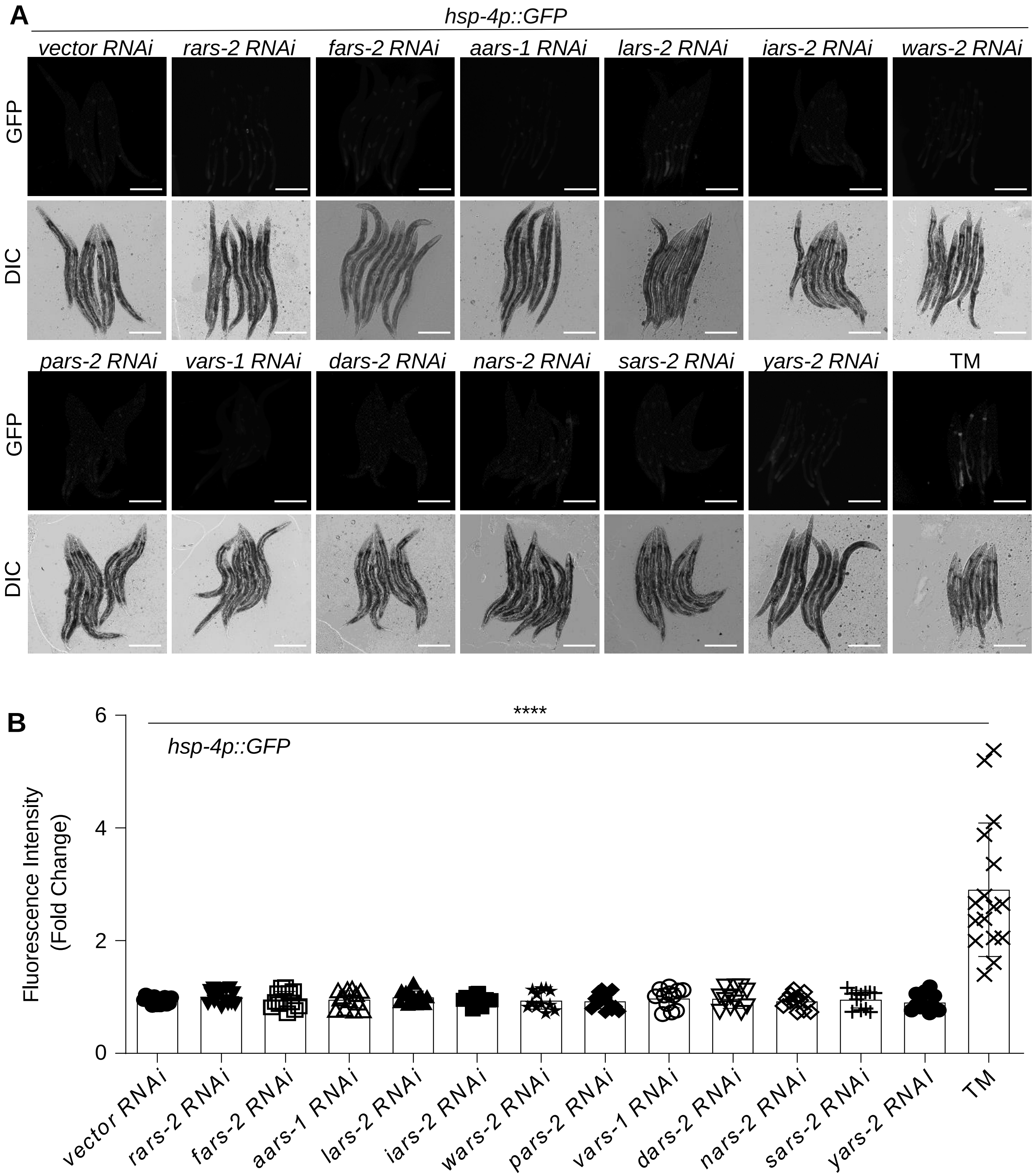

Figure S4 Inactivation of mitochondrial aminoacyl-tRNA synthetase genes does not activate UPR^{ER}. Related to Figure 6.

All worms carry a *gst-4p::GFP* transgenic reporter for UPR^{ER}. (A) Representative images. (B) Quantification graph. As a positive control, treatment with tunicamycin (TM) for 2 hr induced UPR^{ER}. Scale bars, 300 µm. n=14~15. Error bars represent standard error of mean. n.s, indicates no significant difference. ****P<0.0001 (ANOVA with Dunnett's test)

Figure S5

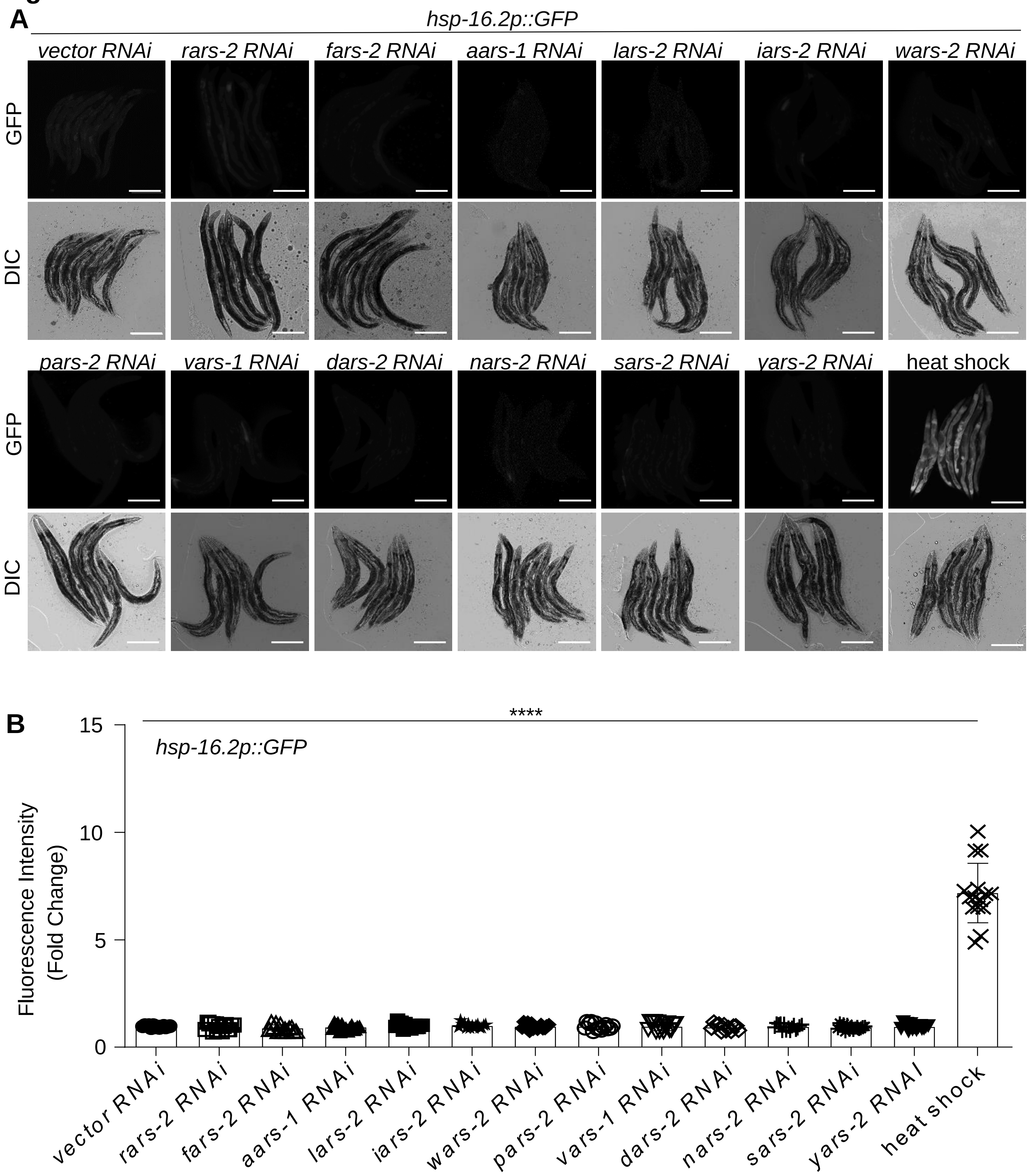

Figure S5 Inactivation of mitochondrial aminoacyl-tRNA synthetase genes does not activate heat shock response. Related to Figure 6.

All worms carry a *hsp-16.2p::GFP* transgenic reporter for heat shock response. (A) Representative images. (B) Quantification graph. As a positive control, heat shock for 1 hr at 33 °C induced heat shock response. Scale bars, 300 μm. n=14~15. Error bars represent standard error of mean. n.s, indicates no significant difference. ****P<0.0001 (ANOVA with Dunnett's test)

Figure S6

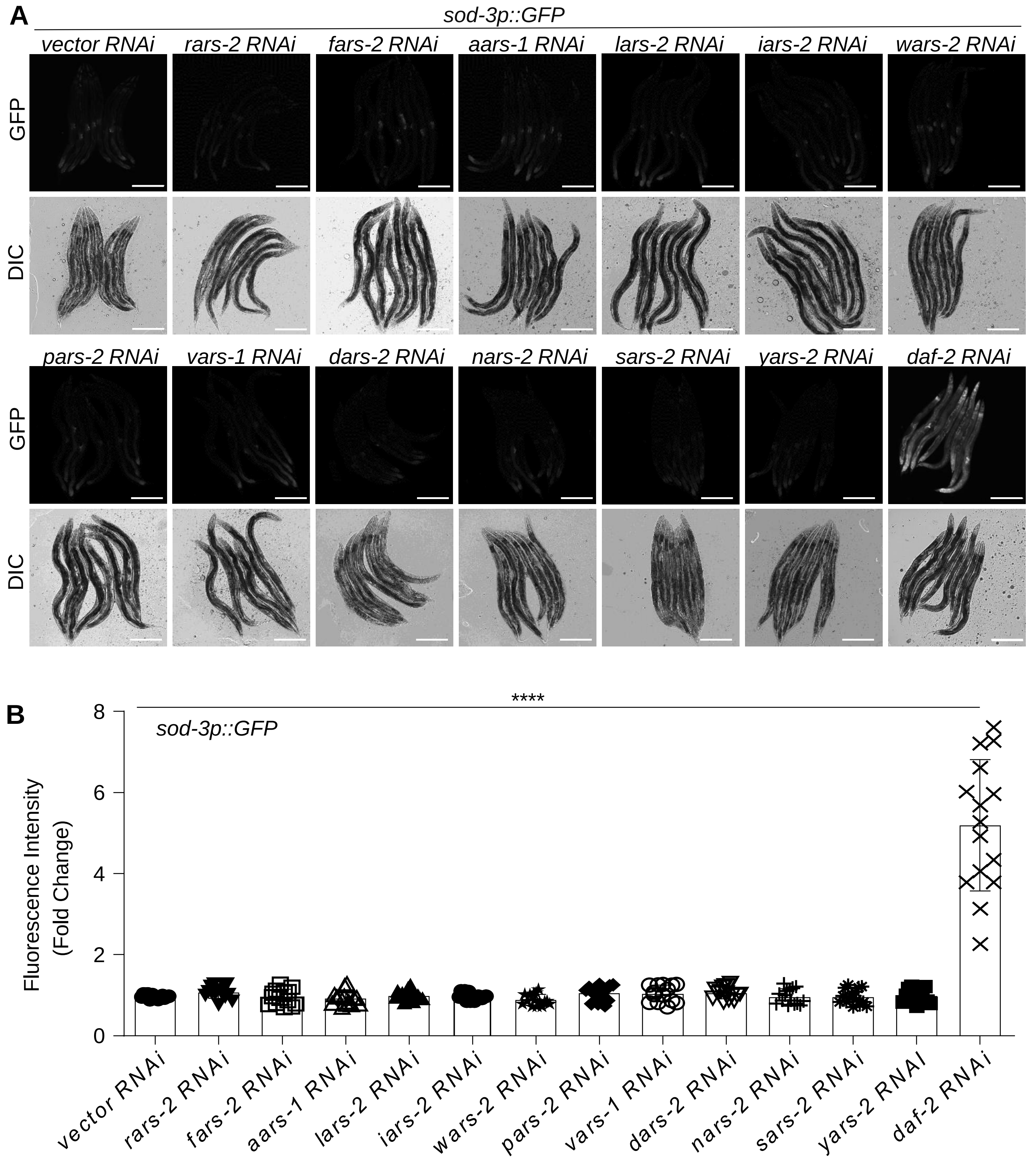

Figure S6 Inactivation of mitochondrial aminoacyl-tRNA synthetase genes does not activate DAF-16. Related to Figure 6.

All worms carry *a sod-3p::GFP* transgene reporting the activity of *daf-16*. (A) Representative images. (B) Quantification graph. As a positive control, *daf-2* RNAi upregulated the expression level of *sod-3p::GFP*. Scale bars, 300 μm. n=14~15. Error bars represent standard error of mean. n.s, indicates no significant difference. ****P<0.0001 (ANOVA with Dunnett's test)

Figure S7

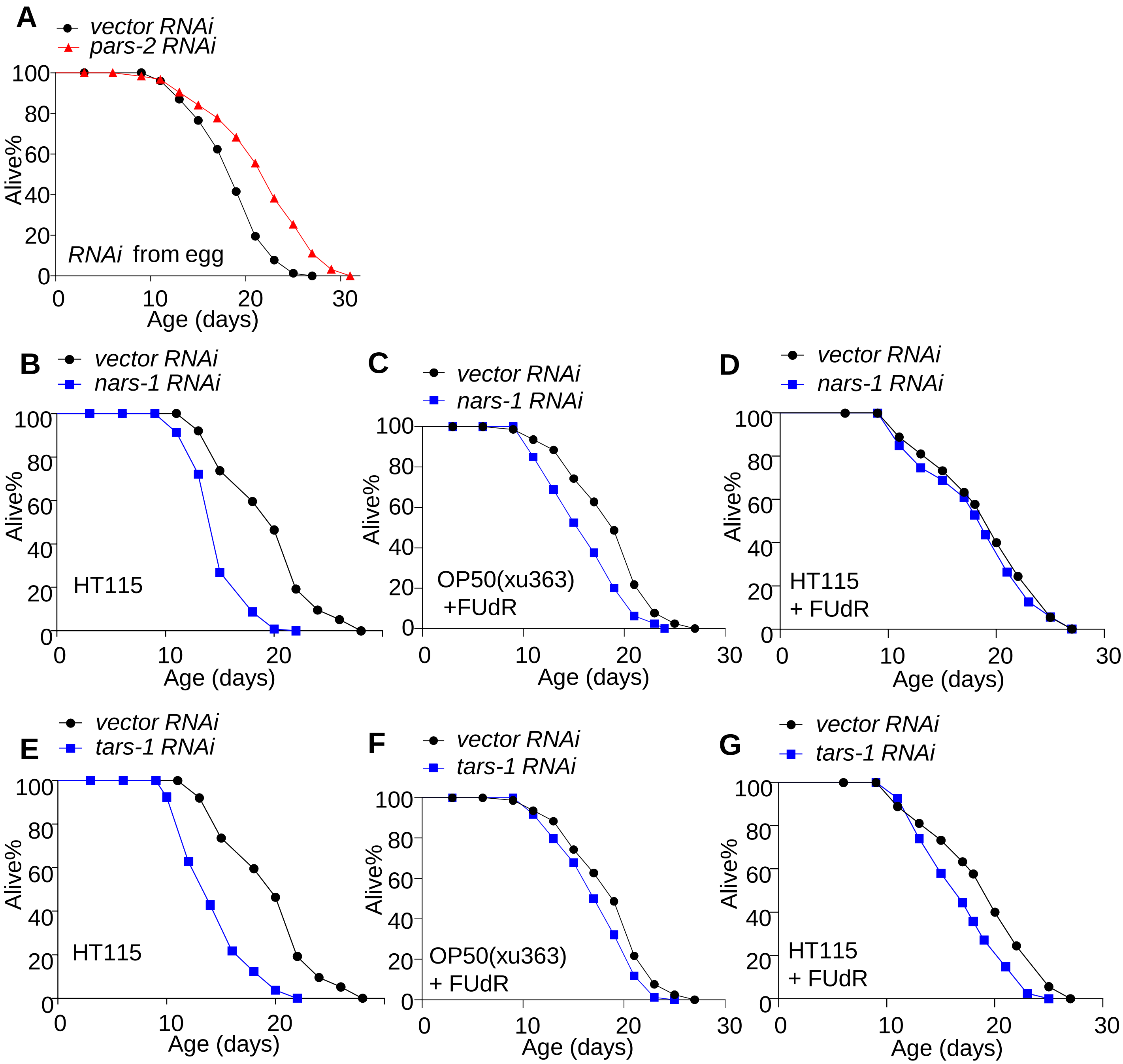

Figure S7 Additional lifespan data from some ARS genes under different RNAi conditions. Related to Figure 7.

(A) *pars-2* RNAi from egg stage extends lifespan.

(B-D) *nars-1* RNAi using HT115 bacteria with (D) or without (B) 5-Fluoro-2'-deoxyuridine (FUdR), or using OP50(xu363) bacteria with FUdR (C) shorten lifespan.

(E-G) *tars-1* RNAi using HT115 bacteria with (G) or without (E) 5-Fluoro-2'-deoxyuridine (FUdR), or using OP50(xu363) bacteria with FUdR (F) shorten lifespan.

B and E share the same vector RNAi curve as they were done at the same time. C and F share the same vector RNAi curve as they were done at the same time. D and G share the same vector RNAi curve as they were done at the same time. All lifespan assays were performed at 20°C and repeated at least twice. Log-rank (Kaplan-Meier) was used to calculate *P* values.

Table S2 Primers of RNAi plasmids. Related to STAR Methods.

gene	primers
hars-1	Forward: 5'-GACTAGTAACCTGTAATGTCTCGTGGA-3'
	Reverse: 5'-CGACGCGTTTGCGTTAACAATTTCGGAT-3'
rars-1	Forward: 5'-GCTCTAGAGATGCCAAGAAATTGAACGA-3'
	Reverse: 5'-GGGGTACCATATGTGAATCCACCGTCA-3'
rars-2	Forward: 5'-CATGCCATGGAGATCAAGCAATTAACCGAT-3'
	Reverse: 5'-GGGGTACCTGCCTTTTCCAAGTCGTTC-3'
kars-1	Forward: 5'-TCCCCGCGGTTTCTGAGCCAAAACACTCG-3'
	Reverse: 5'-GACTAGTTCTCCAGCATCCTTGTCC-3'
fars-1	Forward: 5'-CATGCCATGGCCAGCAATGGAATCTCGA-3'
	Reverse: 5'-GGGGTACCAGTTATATCCAGCGGAACCA-3'
fars-2	Forward: 5'-CATGCCATGGTCCAGACGCATTATACAACC-3'
	Reverse: 5'-GGGGTACCGATAATGTGTCCGATCGACT-3'
fars-3	Forward: 5'-CATGCCATGGGACATTCCAGCAAATCGCTA-3'
	Reverse: 5'-GGGGTACCCGGCTTCGATGTAGTATCCA-3'
aars-1	Forward: 5'-CATGCCATGGACCGAATCTAGAAGAGTTGC-3'
	Reverse: 5'-GGGGTACCTCGAATTCCGTTTCATCGAC-3'
aars-2	Forward: 5'-CATGCCATGGCGGAATCGCTCTCGTGGT-3'
	Reverse: 5'-GGGGTACCAAGAGAACACGTCCACGAGA-3'
lars-1	Forward: 5'-CATGCCATGGGGCTATGAAACATCCCGAAC-3'
	Reverse: 5'-GGGGTACCTTTCCACCTTTCTTCCCGAT-3'
lars-2	Forward: 5'-CATGCCATGGTCTCGCAAAATACGAAAGAGT-3'
	Reverse: 5'-GGGGTACCACTCTTTCGTATTTTGCGAGA-3'
mars-1(let-65)	Forward: 5'-GCTCTAGACCCGTTATTGTAATCTACGAG-3'
	Reverse: 5'-GGGGTACCTTAAGATAACAAATCGGGAA-3'
iars-1	Forward: 5'-GACTAGTTGTCAAAAGATCGTCCAC-3'
	Reverse: 5'-CGACGCGTTCTCTAAGCCAGTTAGCAA-3'
iars-2	Forward: 5'-GACTAGTTCTTTCCTCGTCATCCAC-3'
	Reverse: 5'-CGACGCGTGTTCCAACTGTATCCGTA-3'
wars-1	Forward: 5'-GACTAGTTCCAGCCGAACAAGTTGC-3'
	Reverse: 5'-CGACGCGTTTCAATCTTGGAGCCACGTC-3'
pars-1	Forward: 5'-GCTCTAGACTAAGGCAGAAATGATCGAA-3'
	Reverse: 5'-GGGGTACCCAGTATAATGCACTTATCGT-3'
pars-2	Forward: 5'-CATGCCATGGACTGATCGCAACACTATCACC-3'
	Reverse: 5'-GGGGTACCCTCCGCTCCGTCTCGTTC-3'
vars-1	Forward: 5'-GACTAGTTCACGCAGGAATCGCCACA-3'
	Reverse: 5'-CGACGCGTAGCTATCATTCGTGCAACCC-3'
vars-2(glp-4)	Forward: 5'-CATGCCATGGCCGTCGAGGATACTATCACC-3'
	Reverse: 5'-GGGGTACCATAGCTGCCTAGAGATACACC-3'
cars-1	Forward: 5'-CATGCCATGGTACGAAGCCATCACC-3'
	Reverse: 5'-GGGGTACCACTGGCAGATGTTATCA-3'
gars-1	Forward: 5'-CATGCCATGGTCTACGCTCGGCTTC-3'
	Reverse: 5'-GGGGTACCAAACGTAACCGCTCCG-3'
qars-1	Forward: 5'-GCTCTAGATCCAAGTGCCTGATTTCGAG-3'
	Reverse: 5'-GGGGTACCAAGCTGCTGAAAATTATCCG-3'
nars-1	Forward: 5'-CATGCCATGGTGATCAGCATCCCGGAA-3'
	Reverse: 5'-GGGGTACCAACAAATAACATCCCGTGTCAGCA-3'
nars-2	Forward: 5'-CATGCCATGGATTACGGAAAATTTGGTTT-3'
	Reverse: 5'-GGGGTACCAGAGCTAGACGCTAAAA-3'

Table S2 Primers of RNAi plasmids (continued). Related to STAR Methods.

gene	primers
sars-1	Forward: 5’-CATGCCATGGCATTGACATGTTCCGTA-3’
	Reverse: 5’-GGGGTACCAAGGAGCTTTTCTTCGTTT-3’
sars-2	Forward: 5’-CATGCCATGGGCTGGAGAAGAATCACA-3’
	Reverse: 5’-GGGGTACCACGCTTTAGTGAACAA-3’
yars-1	Forward: 5’-CATGCCATGGATCACTTGGCGTT-3’
	Reverse: 5’-GGGGTACCAACCTCGCCTTTTAAC-3’
yars-2	Forward: 5’-CATGCCATGGTTCGTGGATTACATCACC-3’
	Reverse: 5’-GGGGTACCACTACGCCACATGTTTTC-3’
tars-1	Forward: 5’-CATGCCATGGGGCCCCAACCGAT-3’
	Reverse: 5’-GGGGTACCACTCTGAAGGCCAGTAAC-3’
dars-1	Forward: 5’-CATGCCATGGTTTGTTAACTATGCTCGT-3’
	Reverse: 5’-GGGGTACCATGCTCCAAACATTAATATC-3’
dars-2	Forward: 5’-CATGCCATGGATCTTGTATTTAGCGTTC-3’
	Reverse: 5’-GGGGTACCATAGGCAGTGTCTTTTCT-3’
ears-1	Forward: 5’-CATGCCATGGATAACGAAAATCAGTGCAAC-3’
	Reverse: 5’-GGGGTACCAATTTGACCTGCAATATCGG-3’
ears-2	Forward: 5’-CATGCCATGGTTATGAAGCTAACCGGAT-3’
	Reverse: 5’-GGGGTACCAACCTGCTTTACTAACTTG-3’

Table S3 Primers of confocal micro-injection plasmids. Related to STAR Methods.

Plasmid name	primers
PBS77::ears-2p::ears-2 (cDNA)::mCherry2	Forward:5’-CCCGGGTCTTTATCCTTTCCGATAGT-3’ (promoter)<br>Reverse: 5’-CCTGCAGGCATAATTTCTATTGAGGGGT GCGGTT-3’ (promoter)<br>Forward: 5’-GAGGACCCTTGAGGGTACCATGTCATC TAAAATGACCGCC-3’ (cDNA)<br>Reverse: 5’-ACCCTTTGAGACCATGGTACCGGATCC ACCACCACCAGATTATAATAATTCCTGCATTA-3’ (cDNA)
PBS77::myo-3p::ears-1(cDNA)::mCherry2	Forward: 5’-GAGGACCCTTGAGGGTACCATGATGAT CACCAGAGAGTTGG-3’ (cDNA)<br>Reverse: 5’-ACCCTTTGAGACCATGGTACCGGATCCA CCACCACCAGATTACTTCTTCTTTCCTTTC-3’ (cDNA)
PBS77::myo-3p::aars-1(cDNA)::mCherry2	Forward: 5’-GAGGACCCTTGAGGGTACCATGGGAAT TGGCTCCAAAATA-3’ (cDNA)<br>Reverse: 5’-ACCCTTTGAGACCATGGTACCGGATC CACCACCACCAGATTATTTCTTCTGTTTCAGCA-3’ (cDNA)
PBS77::myo-3p::aars-2(cDNA)::mCherry2	Forward: 5’-GAGGACCCTTGAGGGTACCATGAAGCA CCTGACTGCCTCA-3’ (cDNA)<br>Reverse: 5’-ACCCTTTGAGACCATGGTACCGGATC CACCACCACCAGATTAATTGATTGCAGCAAGGG-3’ (cDNA)
PBS77::myo-3p::vars-1(cDNA)::mCherry2	Forward: 5’-CAGGAGGACCCTTGAGATGGGTAAATT GAATCGATTAGCAC-3’ (cDNA)<br>Reverse: 5’-CTTCACCCTTTGAGACCATGGATCCAC CACCACCAGAACTTTGATTCAAAATTAGCTGTTTAATCCGTT CAGC-3’ (cDNA)
PBS77::myo-3p::vars-2(cDNA)::mCherry2	Forward: 5’-GAGGACCCTTGAGGGTACCATGTCGGA CCCCGCAGGAAA-3’ (cDNA)<br>Reverse: 5’-ACCCTTTGAGACCATGGTACCGGATC CACCACCACCAGATTAGTTGAGAGCCTTGAGTT-3’ (cDNA)
